# Supplementary material for: A competitive protein interaction network buffers Oct4-mediated differentiation to promote pluripotency in embryonic stem cells
Source: Mol Syst Biol. 2013 Oct 8;9:694. doi: 10.1038/msb.2013.49 (PMC3817399; doi:10.1038/msb.2013.49)

Supplementary Material

Table of contents:

- Supplementary information:
  - *A transcriptional circuit fails to reproduce the observed protein correlations associated with pluripotency*
  - *Protein degradation contributes to the heterogeneity of an ES cell population*
  - *Measurements of protein stabilities (half-lives)*
  - *A minimal competitive protein interaction network: The NOC model*
  - *Generalisation of the NOC model to multimeric interactions*
  - *The TBON model*
  - *Parameter sensitivity analysis*
  - *Effects of Chiron and PD03 on the activity of the network*
  - *Bioinformatics analysis of Oct4 and Nanog targets*
  - *Simulation and results of the behavior of the network in different growth conditions and in mutants for its components elements.*
  - *Simulation of the decay of the TBON elements after cyclohexamide treatment*
  - *Effect of a self-repressive feedback loop on Nanog*
- Supplementary figure legends
- Supplementary references
- Supplementary figures S1-S18

A transcriptional circuit fails to reproduce the observed protein correlations associated with pluripotency

A number of screens and functional studies over the last few years have gathered a large amount of information on the transcriptional control of pluripotency (Campbell et al, 2007; Kim et al, 2008; Loh et al, 2006; Lu et al, 2009; MacArthur et al, 2012; Martello et al, 2012; Matoba et al, 2006; Nguyen et al, 2009; Nishiyama et al, 2013; Nishiyama et al, 2009; Sharov et al, 2008; Sharov et al, 2011; Tam et al, 2008; Yi et al, 2008). Functional studies of the networks that can be generated from these reports highlight a small number of transcription factors (Oct4, Nanog, Sox2, Esrrb and Tcf3) that can be assembled in a core network maintaining pluripotency (reviewed in (Chambers & Tomlinson, 2009; Young, 2011), and shown in Figure S1A). Experiments support the notion that this network forms a hub for pluripotency and regulates a number of secondary networks.

The edges of the network in Figure do not necessarily represent simple direct regulatory events, and are based on reproducible observations. Two transcription factors, Oct4 and Sox2, activate each other and both regulate Nanog positively (Chew et al, 2005; Masui et al, 2007; Rodda et al, 2005; Sharov et al, 2008). In turn, Nanog activates Esrrb expression (Festuccia et al, 2012), and represses its own transcription (Navarro et al, 2012); this leads to an indirect feedback on Oct4, as Essrb participates in Oct4 autoregulation (Zhang et al, 2008). Oct4 activates the expression of Tcf3, though how direct is this event is a matter for discussion (Sharov et al, 2008; Yi et al, 2008). Tcf3, finally, represses both Nanog and Esrrb (Martello et al, 2012; Pereira et al, 2006).

We have used this regulatory network to test whether a transcriptional set of interactions alone are able to describe the correlation profiles between Oct4 and Nanog proteins observed experimentally. In order to do this we simplify the circuit by making several experimentally supported assumptions:

- We consider that Oct4 and Sox2 behave as a single element (green region in Fig. S1A), since they positively regulate each other and thus can be expected to have similar activity within the network (Chew et al, 2005; Rodda et al, 2005).
- We can also bind Oct4 and Esrrb together (blue region in Fig. S1A) because of their similar effect on Nanog and of the observation that they operate together in this activity (van den Berg et al, 2010; van den Berg et al, 2008).
- Finally, we ignore the negative feedback of Oct4 on itself via Tcf3, and the direct auto-repression of Nanog (Navarro et al, 2012). These simplifications rely on the observation that negative feedbacks promote homeostasis (Alon, 2007; Becskei & Serrano, 2000; Savageau, 1974), and thus are not expected to affect correlations between pairs of

molecules in an asymmetric way, as we experimentally observe in this study. In agreement with this, adding the negative feedback of Nanog does not change qualitatively the behaviour of our protein-interaction network, as shown in Figure S18 using the extended TBON model (see below).

As a result of these simplifications, we are left with the transcriptional regulatory network depicted in Figure S1B, in which Oct4, Esrrb and Sox2 are combined into a single dynamical node whose interactions with the other circuit elements (Nanog and Tcf3) correspond to the sum of all interactions affecting all three proteins. This network is topologically equivalent to the one we have discussed before and which can account for the fluctuations of Nanog expression (Kalmar et al, 2009). Assuming that the dynamics of Tcf3 adapts instantaneously to the levels of the other two proteins (adiabatic elimination), the dynamics of the circuit shown in Figure S1B is described by the following two-dimensional differential-equation model:

$$\frac{dN}{dt} = \alpha_n + \frac{\beta_n N^n}{k_n^n + N^n + (gA)^m} - \gamma_n N,$$

$$\frac{dA}{dt} = \alpha_a + \beta_a AN - \gamma_a A,$$

with $\alpha_n = 0.15$ prot h^{-1}, $\beta_n = 3.75$ prot h^{-1}, $\gamma_n = 7 \cdot 10^{-6}$ h^{-1}, $k_n = 4000$ prot, $g = 1$, $n = 2$ $m = 2$, $\alpha_a = 5 \cdot 10^{-4}$ prot h^{-1}, $\beta_a = 9 \cdot 10^{-10}$prot h^{-1} and $\gamma_a = 5 \cdot 10^{-4}$h^{-1}. Here N represents the level of Nanog and A stands for the joint action of Oct4, Esrrb and Sox2. In this model, the regulated expression of Nanog is assumed to be cooperative (both for N and A) and subject to saturation. On the other hand, the transcriptional input on A is considered to be linear in N and to depend on A itself (in the form of a positive feedback that accounts for the mutual activation between Oct4 and Sox2), and saturation is ignored.

The typical behavior of this model is shown in Figure S1C, which plots the state of the system for increasing time in the $N - A$ plane, as obtained from stochastic simulations, by means of dots, and the nullclines of the deterministic equations in blue and green lines. For the parameters chosen, the system has one single stable state that corresponds to high concentrations of Nanog and Oct4/Esrrb/Sox2. Sporadic transcriptional fluctuations might perturb the stable state and trigger a dynamic response that leads to a sudden decrease of Nanog followed by a slow decrease of Oct4/Esrrb/Sox2 levels. These decreases alleviate repression of the Nanog promoter; when Nanog levels are recovered so are the levels of Oct4/Esrrb/Sox2, thus returning the system to the high-Nanog steady state. Such a dynamic response, known as *excitability* (Rue & Garcia-Ojalvo, 2011), creates a trajectory in the $N - A$ phase space in the form of cycles, with Nanog and Oct4 levels correlated in a highly

nonlinear manner, thus leading to a joint distribution with a very small linear correlation coefficient, far away from the experimental observations (Fig. 1A). Indeed, the results shown in Fig. S1C are qualitatively independent on the specific functional forms considered for the interactions between the circuit elements, although they quantitatively depend on the shape of the non-linear terms involved in the reaction rates, and, more specifically on the shape of the nullclines.

Thus we conclude that, on its own, the proposed transcriptional network cannot reproduce the correlations at the level of protein that we observe in single cells associated with pluripotency.

A recent discussion of transcriptional networks has suggested situations in which a transcriptional network can lead to correlated levels of protein expression of its component elements (Munsky et al, 2012). For a case of two elements (Nanog and Oct4 in our case) such a correlation would require the coordinated expression of both mRNAs upon regulation by a third protein. In other words, the mRNAs of both elements should have the same kinetics. In our case, this would imply that Oct4 and Nanog mRNAs show the same distribution at the single cell level. However this is not observed: while in S+L conditions most of the mES cells express Oct4, only a subset of them express Nanog (Hayashi et al, 2008; Trott et al, 2012). While the transcriptional model discussed above is not comprehensive, it gives an insight into the limitations of transcription regulation models and their inability to reproduce the observed correlations of protein expression from a set of transcriptional interactions alone.

Protein degradation contributes to the heterogeneity of an ES cell population

The relevance of considering protein stabilities in the maintenance of pluripotency is supported by the effect that proteasome inhibition has on Nanog and Oct4 (and also β-catenin) distribution (Fig. S2). Stopping protein degradation for just 2 h with the proteasome inhibitor MG132 results in increased Nanog, Oct4 and β-catenin, and also a decreased heterogeneity in their levels. These results are similar to culturing mES cell in 2i+L conditions (compare with Fig. S6A-C).

Measurements of protein stabilities (half-lives)

The average stability of the proteins can be estimated experimentally by measuring the half-life in the presence of cyclohexamide (CHX), which stops protein translation. We have measured the outcome of this perturbation in two ways: through analysis of Western Blots

(WB) of total cell lysates, and also through quantitative immunofluorescence (QIF) analysis (Figure S3B-K). The WB results show that while total β-catenin is part of a very stable protein pool, Oct4 and Tcf3 are less stable, and Nanog is very unstable (Figure S3B). Similar results were obtained using QIF to estimate the rates of degradation of Oct4, Nanog, β-catenin and Tcf3 in populations of single cells (Fig. S3C-F, K). QIF provides additional information that is not available in the average measurements characteristic of WBs, e.g. how the distribution of the cells in the scatterplots evolves after stopping protein production. The average immunofluorescence of the population at different times after cyclohexamide treatment reveals an average half-life for the proteins that is consistent with that obtained from Western blots of whole populations, thereby validating the use of this technique. Our estimates for Nanog and Oct4 proteins half-lives are in agreement with independent reports (Abranches et al, 2013)

Fitting these results with a linear decay model (Figure S3G-J show fittings to WB data) confirms that the kinetics of decay is consistent with a hierarchy of protein stabilities in which Nanog is the most unstable element, then Oct4, and finally Tcf3 and β-catenin. This information is fed into the models developed below.

A minimal competitive protein interaction network: The NOC model

To account for the observed distributions of Nanog and Oct4 at the level of single cells, we first developed a simple mathematical model of the core post-transcriptional interactions between those two proteins. The model describes the dynamics of the concentrations of Nanog (N) and Oct4 (O), which interact to form a complex (hereafter denoted by O:N) (Fig. 1F). Thus, the total amounts of Nanog and Oct4 in the system are given by $N_t = N + O{:}N$, $O_t = O + O{:}N$, respectively. The deterministic equations read:

$$\frac{dN}{dt} = k_{-} O{:}N - (k_{+} O + \lambda_N) N,$$
$$\frac{dO}{dt} = k_{-} O{:}N - (k_{+} N + \lambda_O) O,$$
$$\frac{dO{:}N}{dt} = k_{+} N \cdot O - (k_{-} + \lambda_{ON}) O{:}N.$$

The Nanog-Oct4 complex association and dissociation processes are represented by mass action kinetics with rates $k_{+}$and k_{-}, respectively. These terms assume, for simplicity, monomeric participation of both proteins in the complex, although the behaviour of the model is qualitatively the same with multimeric interaction (see below). Finally, protein and complex degradation is taken into account through linear decay of Nanog, Oct4 and the complex with rates λ_N, $\lambda_O$and λ_{ON}, respectively.

A key assumption of this model concerns the different stabilities for the free proteins and protein complexes. Specifically, free Nanog is assumed to decay much faster than Oct4 and the complex ($\lambda_N \gg \lambda_O, \lambda_{ON}$), as suggested from the protein half-life measurements showing that Nanog is less stable than Oct4 (see Fig. S3). Intuitively, unstable free Nanog implies that the bulk of Nanog proteins will be in a complex with Oct4 and thus, loosely speaking, the system requires minimally one molecule of Oct4 for each molecule of stabilized Nanog. This provides a mechanism responsible for generation of the sharp boundary in the Nanog-Oct4 distribution (Figure 1D in the main text and (Munoz Descalzo et al, 2012)). Figure S4G shows several trajectories in the state space formed by free Nanog-free Oct4-O:N complex, corresponding to the response of the continuous model given above to pulses of Nanog and Oct4 production of different amplitudes. All these trajectories have an initial transient where free Nanog (N) rapidly disappears in two different ways: i) if there is free Oct4 available, Nanog binds to it; otherwise ii) it quickly degrades. As a result, all trajectories collapse onto the zero-N plane and then Oct4 and O:N slowly decay to zero. If we translate the free variables of the system (N, O, O:N) to the measurable variables N_t and O_t, as shown in Figure S4H, all trajectories collapse to the line $N_t = O_t$. However, trajectories below this line collapse much faster than those above it. Thus, this line defines the O:N boundary observed in the experiments.

In order to account for fluctuations in the expression of the Nanog and Oct4 genes, we have implemented a discrete stochastic version of the model. The transcriptional layer, for which no regulation is assumed, introduces noise in the network of protein interactions. The expression of Nanog and Oct4 is modelled with two alleles each, which can be in the active or inactive states, thus allowing for different transcriptional patterns which range from bursty infrequent expression for Nanog in S+L conditions to its uninterrupted expression in 2i+L, depending on the values of the state-switching rates. The full set of simplified reactions reads

Transcriptional layer ($\alpha = 1,2$)

$$P_{N,0}^{\alpha} \xrightarrow{k_{+1}} P_{N,1}^{\alpha} \qquad P_{N,1}^{\alpha} \xrightarrow{k_{-1}} P_{N,0}^{\alpha}$$

$$P_{O,0}^{\alpha} \xrightarrow{k_{+2}} P_{O,1}^{\alpha} \qquad P_{O,1}^{\alpha} \xrightarrow{k_{-2}} P_{O,0}^{\alpha}$$

$$P_{N,1}^{\alpha} \xrightarrow{k_3} P_{N,1}^{\alpha} + mN \qquad P_{O,1}^{\alpha} \xrightarrow{k_4} P_{O,1}^{\alpha} + mO$$

$$mN \xrightarrow{k_5} mN + N \qquad mO \xrightarrow{k_6} mO + O$$

$$mN \xrightarrow{k_7} \varnothing \qquad mO \xrightarrow{k_8} \varnothing$$

Post-translational layer

$N \xrightarrow{k_9} \emptyset$ $\quad O \xrightarrow{k_{10}} \emptyset$ $\quad ON \xrightarrow{k_{11}} \emptyset$

$N + O \xrightarrow{k_{+12}} ON$ $\quad ON \xrightarrow{k_{-12}} N + O$

Here $P_{i,j}^{\alpha}$ represent the inactive ($j = 0$) and active ($j = 1$) states of the promoters of the two alleles ($\alpha = 1,2$) of Nanog ($i = N$) and Oct4 ($i = O$). The Nanog and Oct4 promoters randomly switch their states with transition rates $k_{\pm 1}$ and $k_{\pm 2}$. When in the active state, the promoters generate transcripts at rates k_3 and k_4, and are assumed to decay with half-lives of $\ln 2/k_7$ and $\ln 2/k_8$, respectively (Fig. S4A). Nanog and Oct4 transcripts are then translated at rates k_5 and k_6, feeding the post-translational network of interactions depicted in Figure 1F of the main text, and implemented by the last 5 reactions in the discrete scheme above. Only these last 5 interactions are responsible of the correlations, and do not feed back to the unregulated transcriptional layer. The simulations of the NOC model shown in this paper are performed with an implementation of the Gillespie algorithm using the parameter values in Table SI. Parameter values of the mRNA and protein degradation are based on experimentally estimated half-lives (cf. Table SI and Fig. S3A,K).

In the framework of the discrete model outlined above, the different culture conditions (S+L and 2i+L) are represented by the Nanog expression level. In particular, following recent experimental results ((Miyanari & Torres-Padilla, 2012; Navarro et al, 2012) and Fig. 1H, I) standard self-renewing (S+L) conditions are implemented by infrequent transcriptional bursts of Nanog expression (Figure S4A), while 2i+L conditions are modelled by substantially increasing of the frequency of the transcription events (Table SI and Figure S4B). This simple model is able to reproduce Nanog mRNA and protein distribution obtained experimentally in S+L and 2i+L conditions (compare Fig. S4C, E with Figs. 1I and S6A, respectively) and, to a certain extent, that of Oct4 levels (compare Fig. S4F with Fig. S6B).

According to the model, the joint effect of continuous Oct4 expression with bursty Nanog transcription and the higher stability of Oct4 and O:N allows the system to explore the space above the boundary in S+L conditions (Fig. 1D,G). In 2i+L conditions, on the other hand, continuous expression of Nanog leads to uniform distribution of Nanog proteins (Fig. S4E), which are highly correlated to levels of Oct4 (Fig. 1E,G).

To further assess the role of bursty Nanog expression in S+L conditions, we investigated the dynamics of Nanog expression *in silico*, using the NOC model. In this model, burstiness can be controlled without affecting the average levels of Nanog mRNA. This is achieved by modifying the mean residence-times in the active and inactive states of the Nanog promoters without modifying the ratio of time that the promoters are in the active state, i.e. a timescale parameter ϕ controls the activation and inactivation transition rates $k_{+1}^{\phi} = \phi \cdot k_{+1}$ and

$k_{-1}^{\phi} = \phi \cdot k_{-1}$. The upper and middle panels in Fig. S4I show that the parameter ϕ does not affect the ratio of promoter activity nor the average number of mRNA molecules. This parameter, however, does have a clear impact on the O/N correlation, as can be observed in the bottom panel in Fig. S4I: the slower the transition rates, the lowest the correlation achieved, as the system is allowed to remain without Nanog production enough time to become completely depleted (cf. Figs. S4K and J). Conversely, the faster (and less bursty) the Nanog promoter is, the higher O/N correlation is achieved, as in the case of 2i+L conditions (cf. Figs. S4K and L).

Param.	Value	Units	Description
k_{+1}	0.04 (S+L)<br>0.1 (2i+L)	h^{-1}	Nanog promoter activation rate
k_{-1}	1	h^{-1}	Nanog promoter inactivation rate
k_{+2}	0.1	h^{-1}	Oct4 promoter activation rate
k_{-2}	1	h^{-1}	Oct4 promoter inactivation rate
k_3	25	trans h^{-1}	Nanog mRNA transcription rate
k_4	25	trans h^{-1}	Oct4 mRNA transcription rate
k_5	100	prot trans^{-1}h^{-1}	Nanog translation rate
k_6	25	prot trans^{-1}h^{-1}	Oct4 translation rate
k_7	0.1	h^{-1}	Nanog mRNA degradation rate
k_8	0.1	h^{-1}	Oct4 mRNA degradation rate
k_9 (λ_N)	5	h^{-1}	Free Nanog degradation rate
k_{10} (λ_O)	0.1	h^{-1}	Free Oct4 degradation rate
k_{11} (λ_C)	0.1	h^{-1}	Nanog-Oct4 complex degradation rate
k_{+12} (k_+)	0.2	prot^{-1}h^{-1}	Nanog-Oct4 complex association rate
k_{-12} (k_-)	0.5	h^{-1}	Nanog-Oct4 complex dissociation rate

Table SI: Parameter values for the NOC model

Generalisation of the NOC model to multimeric interactions

The minimal NOC model assumes direct interaction between one Nanog molecule and one Oct4 molecule. However, this interaction can be easily extended to multimeric species, which have been reported (Carey et al, 2011; Mullin et al, 2008; Saxe et al, 2009). If, for instance, the interaction is accomplished through a complex involving a Nanog *N*-mer and an Oct4 *M*-mer, the correlations will not change drastically provided the homomeric complexes are as unstable as their simple protein counterparts. What is expected to change is the ratio Oct4 to Nanog, which will tend to the rate *M*/*N*. To illustrate this generalisation of the NOC model,

we simulated the cases in which either both Nanog and Oct4 dimerize prior to interact (Fig. S5A,C), or Nanog forms a dimer and then is complexed with Oct4 (Fig. S5B,C).

The TBON model

In order to incorporate the effects of Tcf3 (T) and β-catenin (β) on the regulation of the pluripotency state, we developed an expanded protein interaction network model that extends the concept of stable complexes to those two proteins, and also includes the effect that culturing cells under 2i+L conditions has on their behaviour. In this model, we consider three binary protein complexes: Nanog and Oct4 are assumed to interact in a complex, as in NOC (O:N, (Fidalgo et al, 2012; Liang et al, 2008; van den Berg et al, 2010; Wang et al, 2006; Zhang et al, 2007)); β-catenin interacts with Oct4 (β:O, (Abu-Remaileh et al, 2010; Ding et al, 2012; Faunes et al, 2013; Kelly et al, 2011; Takao et al, 2007)) and β-catenin interacts, also with Tcf3 (β:T, (Shy et al, 2013; Yi et al, 2011)). Experimentally, we observe that the β-catenin:Oct4 complex also includes a small amount of Nanog, thus indicating the existence of a ternary complex (β:O:N, Faunes et al. 2013) which we take into account in the model. The outline of the TBON model is shown in Figure 2B of the main text.

Using QIF, we can measure experimentally the total levels of Oct4, Nanog, β-catenin and Tcf3 in single cells (Figure 2A of the main text and S6A-D). Using these measurements as a reference, the model attempts to reproduce the total quantities of protein species, which in this case are $N_t = N + O\!:\!N + \beta\!:\!O\!:\!N$, $O_t = O + O\!:\!N + \beta\!:\!O + \beta\!:\!O\!:\!N$, $\beta_t = \beta + \beta\!:\!O + \beta\!:\!T + \beta\!:\!O\!:\!N$, and $T_t = T + \beta\!:\!T + P_{N,2}^{1} + P_{N,2}^{2}$ (the latter two terms correspond to the Tcf3 molecules that are bound to each of the two alleles of the Nanog promoter in its active state).

As in the NOC model, two alleles (promoters) are considered for Nanog, Oct4 and Tcf3, whereas for β-catenin, the model assumes a constant supply of proteins to the system, which differs from cell to cell according to the observed variability of β-catenin (Fig. 1A in the main text, and S6C). The total amount of β-catenin that is available to the system might be determined from its different pools and their subcellular localization, as has been observed (Faunes et al, 2013). The TBON model concentrates on how the proteins regulate and interact with one another, the only transcriptional regulation included being the inhibition of Nanog by Tcf3 (blue arrow in Fig. 2B).

To model the effect of 2i+L conditions, we used the experimentally observed increase in the levels of β-catenin after Chiron treatment (Faunes et al, 2013; Wray et al, 2011), and the increase in Nanog levels following PD03 treatment, induced by the influence that FGF signalling has on Nanog expression at the transcriptional level (Lanner et al, 2010; Luo et al,

2012; Wray et al, 2011; Wray et al, 2010). Following these observations, we represented the influence of 2i+L conditions as a double effect (red arrows in Figure 2B of the main text): first, Chiron is considered to stabilize β-catenin, which is modelled as a net increase in β-catenin supply rate in the model; second, we consider that PD03 indirectly promotes the continuous expression of Nanog (it activates the promoter and facilitates transcription, Fig. S6K). This is introduced in the model as a parameter change (increase k_{+1}, see Table SII below), which increases the rate of Nanog promoter activation.

A key factor in this model, similarly to the case of the NOC model described above, is the difference in the stability of free Nanog with respect to the complex O:N complex. The set of reactions included in the model is:

Nanog promoters

$$P_{N,0}^{\alpha} \xrightarrow{k_{+1}} P_{N,1}^{\alpha} \qquad P_{N,1}^{\alpha} \xrightarrow{k_{-1}} P_{N,0}^{\alpha}$$

$$P_{N,0}^{\alpha} + T \xrightarrow{k_{+2}} P_{N,2}^{\alpha} \qquad P_{N,2}^{\alpha} \xrightarrow{k_{-2}} P_{N,0}^{\alpha} + T$$

Oct4 promoters

$$P_{O,0}^{\alpha} \xrightarrow{k_{+3}} P_{O,1}^{\alpha} \qquad P_{O,1}^{\alpha} \xrightarrow{k_{-3}} P_{O,0}^{\alpha}$$

Tcf3 promoters

$$P_{T,0}^{\alpha} \xrightarrow{k_{+4}} P_{T,1}^{\alpha} \qquad P_{T,1}^{\alpha} \xrightarrow{k_{-4}} P_{T,0}^{\alpha}$$

mRNA transcription

$$P_{N,1}^{\alpha} \xrightarrow{k_5} P_{N,1}^{\alpha} + mN \qquad P_{O,1}^{\alpha} \xrightarrow{k_6} P_{O,1}^{\alpha} + mO \qquad P_{T,1}^{\alpha} \xrightarrow{k_7} P_{T,1}^{\alpha} + mT$$

Protein translation

$$mN \xrightarrow{k_8} mN + N \qquad mO \xrightarrow{k_9} mO + O \qquad mT \xrightarrow{k_{10}} mT + T$$

mRNA degradation

$$mN \xrightarrow{k_{11}} \varnothing \qquad mO \xrightarrow{k_{12}} \varnothing \qquad mT \xrightarrow{k_{13}} \varnothing$$

β-catenin dynamics (availability of β-catenin to the system)

$$\varnothing \xrightarrow{k_{\beta+}} \beta \qquad \beta \xrightarrow{k_{\beta-}} \varnothing$$

Protein degradation

$$N \xrightarrow{k_{14}} \varnothing \qquad \beta{:}O{:}N \xrightarrow{k_{\beta-}} \varnothing$$

$O \xrightarrow{k_{15}} \varnothing$

$\beta{:}O \xrightarrow{k_{\beta-}} \varnothing$

$T \xrightarrow{k_{16}} \varnothing$

$\beta{:}T \xrightarrow{k_{16}} \varnothing$

$O{:}N \xrightarrow{k_{17}} \varnothing$

Complex formation

$N + O \xrightarrow{k_{+18}} O{:}N$

$O{:}N \xrightarrow{k_{-18}} N + O$

$N + \beta{:}O \xrightarrow{k_{+19}} \beta{:}O{:}N$

$\beta{:}O{:}N \xrightarrow{k_{-19}} N + \beta{:}O$

$O + \beta \xrightarrow{k_{+20}} \beta{:}O$

$\beta{:}O \xrightarrow{k_{-20}} O + \beta$

$T + \beta \xrightarrow{k_{+21}} \beta{:}T$

$\beta{:}T \xrightarrow{k_{-21}} T + \beta$

The parameter values used for the simulation of wild-type cells in S+L conditions are shown in Table SII. Cell to cell variability in β-catenin levels is introduced in the parameter $k_{\beta+}$, which follows a log-normal distribution with mean $\ln \mu_{\beta+}$ and variance $\sigma^2_{\beta+}$. In S+L conditions the values used are $\mu_{\beta+} = 300$ and $\sigma^2_{\beta+} = 0.2$, whereas in 2i+L conditions these are $\mu_{\beta+} = 700$ and $\sigma^2_{\beta+} = 0.6$.

The TBON model thus considers 8 protein species: 4 free proteins (O, N, β and T) and 4 protein complexes (O:N, β:O, β:O:N and β:T). In addition, the transcriptional layer is modelled with 3 mRNA species (mN, mO, mT); and 14 binary variables that indicate the promoter states of each allele of Nanog, Oct4 and Tcf3 ($P^{\alpha}_{N,0}$, $P^{\alpha}_{N,1}$, $P^{\alpha}_{N,2}$, $P^{\alpha}_{O,0}$, $P^{\alpha}_{O,1}$, $P^{\alpha}_{T,0}$ and $P^{\alpha}_{T,1}$, for $\alpha = 1,2$). Although the model requires a total of 32 parameters to be set (Table SII and text above), 15 of these are required for the generation of the observed transcriptional fluctuations (gene transcription states and mRNA dynamics) and their values have been set to experimental estimates (Fig. S3A) or independently fit to transcription data (Fig 1I). The remaining 17 parameters are directly related to the 8 protein species (8 degradation rate constants, 8 association/dissociation rate constants and the rate at which β-catenin is made available to the network). Out of these, the degradation rates are chosen to reproduce the protein half-life data (Fig. S3), and the complex association/dissociation rates are mainly selected to replicate the correlation observations. Thus we have a wide variety of experimental data to constraint the parameter values of our model.

Figures S6A-D show the distributions of Nanog, Oct4, β-catenin and Tcf3 proteins obtained experimentally from mES cells cultured in S+L or 2i+L conditions, while Figs. S6E-H show

their model counterparts. The theoretical mRNA distributions for Nanog and Oct4 are also shown (Fig. S6I,J) as well as the Nanog promoter status (Fig. S6K).

The model provides predictions not only of the distributions of the total amount of protein, but also of its pools of sub-species and complexes. We represent the different pools of each of the proteins by means of the pie charts shown first in Figs. 2F (S+L) and G (2i+L) in the main text (and throughout in subsequent figures). The area of these charts is proportional to the average total amount of each protein in a given condition, which allows a comparison of the total amounts of proteins between different conditions. For instance, Figs. 2F,G show that total Nanog levels in S+L are significantly smaller that in 2i+L. In addition, each circle is split into sectors representing the different pools of protein complexes and free species, with area proportional the corresponding amount of protein. As an example, in standard self-renewal (S+L) conditions, the levels of free Oct4 are much higher than in naïve 2i+L, where these are almost negligible (compare Figs. 2F and G). These pie-chart representations of the protein pools predicted by the model allow a direct comparison between signalling and genetic perturbations of the network.

Because the amount of Nanog in the ternary complex is small, we also considered a model without a complex containing β-catenin and Nanog together; this simpler model reproduces well the experimental observations in Serum and LIF but does not account well for the behaviour in 2i. On the other hand, a model that assumes the existence of a binary complex between β-catenin and Nanog independently of the interaction of β-catenin with Oct4 is largely indistinguishable from the TBON model (data not shown). This is perhaps not surprising, as the predicted amount of Nanog in the ternary complex in S+L is small (3%) and therefore the manner in which it enters the complex should not influence the dynamics of the system. In fact, in such a model the three binary complexes O:N, β:O and β:N would lead to a stationary equilibrium whose time averages matches that of the model with the β:O:N complex.

Parameter	Value	Units	Parameter	Value	Units
$\boldsymbol{k_{+1}}$	2 (S+L)	h^{-1}	k_{11}	0.2	h^{-1}
$\boldsymbol{k_{+1}}$	8 (2i+L)	h^{-1}	k_{12}	0.2	h^{-1}
$\boldsymbol{k_{-1}}$	2	h^{-1}	k_{13}	0.2	h^{-1}
$\boldsymbol{k_{+2}}$	0.005	$trans^{-1}h^{-1}$	k_{14}	6	h^{-1}
$\boldsymbol{k_{-2}}$	2	$trans^{-1}h^{-1}$	k_{15}	0.4	h^{-1}
$\boldsymbol{k_{+3}}$	0.4	h^{-1}	k_{16}	0.15	h^{-1}
$\boldsymbol{k_{-3}}$	2	h^{-1}	k_{17}	0.3	h^{-1}

$\boldsymbol{k_{+4}}$	4	h^{-1}	k_{+18}	0.4	$\mathrm{prot}^{-1}\mathrm{h}^{-1}$
$\boldsymbol{k_{-4}}$	20	h^{-1}	k_{-18}	1	h^{-1}
$\boldsymbol{k_{5}}$	50	trans h^{-1}	k_{+19}	0.0001	$\mathrm{prot}^{-1}\mathrm{h}^{-1}$
$\boldsymbol{k_{6}}$	50	trans h^{-1}	k_{-19}	0.01	h^{-1}
$\boldsymbol{k_{7}}$	50	trans h^{-1}	k_{+20}	100	$\mathrm{prot}^{-1}\mathrm{h}^{-1}$
$\boldsymbol{k_{8}}$	50	prot $\mathrm{trans}^{-1}\mathrm{h}^{-1}$	k_{-20}	0.02	h^{-1}
$\boldsymbol{k_{9}}$	20	prot $\mathrm{trans}^{-1}\mathrm{h}^{-1}$	k_{+21}	20	$\mathrm{prot}^{-1}\mathrm{h}^{-1}$
$\boldsymbol{k_{10}}$	8	prot $\mathrm{trans}^{-1}\mathrm{h}^{-1}$	k_{-21}	0.2	h^{-1}
			$k_{\beta-}$	0.1	h^{-1}

Table SII: Parameter values for the TBON model

Parameter sensitivity analysis

In order to assess the robustness of the model to parameter changes and ensure that it is not overfitted, we performed a sensitivity analysis of the post-translational parameters. We simulated the model with parameter perturbations of ±25% of the original value (taken from SL conditions) and estimated the O/N, B/N and B/O correlations. Figure S7 shows the result of this systematic sensitivity analysis. From these plots it is clear that the correlations are not largely affected by substantial changes in the parameter values. Importantly, the model is largely insensitive to changes in the complex association/dissociation rates. On the other hand, changes in some protein degradation rates, such as the rate of free Oct4 degradation (k_{15}), the rate of free Tcf3 and β:T degradation (k_{16}), or the rate of O:N induce mild changes in the O/N ratio. In all these cases, however, the O/N correlation coefficient remains in the range (0.4, 0.7). Thus the model results do not require an unrealistic overfitting of the experimental observations, while those observations provide us with sufficient constraints to identify the parameter region where the system operates.

Effects of Chiron and PD03 on the activity of the network

The simulations of the TBON allow us to analyse the separate effects that each of the 2i components has on the pluripotent state. To simulate in the effect of Chiron, all parameter values are taken from S+L conditions with the exception of the rate parameters for the production of β-catenin ($\mu_{\beta+}$ and $\sigma^2_{\beta+}$), which are taken from 2i+L conditions, as the amount of β-catenin is the main outcome of the application of Chiron. For the effects of PD03 all parameter values are taken from S+L conditions with the exception of the activation rate of

the Nanog promoter (k_{+1}), which is taken from 2i+L conditions, as the amount of Nanog relies on the release of the repressive effect of ERK.

Notice that Chiron and PD03 have different effects on the core of the protein network: while Chiron increases the levels of β-catenin (as expected), the model suggests that PD03 mildly decreases them (Fig. S8A,B,G,J,K). On the other hand, PD03 induces higher expression of Nanog (since it acts on its promoter) (Fig. S8I), in agreement with experimental observations (Lanner et al, 2010; Miyanari & Torres-Padilla, 2012). The increase and stabilization of Oct4 levels by Chiron (through its action on β-catenin) result in a reduction of the levels of free Oct4 (Fig. S8C,D,J,K). The effect of PD03 is also to reduce the levels of free Oct4, but in this case this is due to the high levels of Nanog that will interact with Oct4 and neutralize its pro-differentiation activity (Fig. S8C,D,J,K). Overall, the different pools of free molecules and complexes vary depending on the relative amounts and stabilities of the complexes, which change from cell to cell, and over time (Fig. S8J,K).

Bioinformatics analysis of Oct4 and Nanog targets

A prediction of the TBON model is that an excess of Oct4 in ES cells is inversely related to their degree of pluripotency (see text for details and Figs. S6L,M and S8C,D). Our working hypothesis is that, when not interacting with Nanog or β-catenin, Oct4 promotes differentiation through the interactions with lineage determinants, and the activation and repression of markers and enforcers of lineage commitment. This notion finds some support in several ES cell expression (Loh & Lim, 2011; Nishiyama et al, 2009; Sharov et al, 2008) and DNA binding (Chen et al, 2008) data sets (see in particular http://www.maayanlab.net/ESCAPE, a database integrating high-throughput data of pluripotency in m/hESCs). In search of further evidence and support of our contention, we performed a comprehensive bioinformatics analysis of publicly available data looking at the genes regulated by Nanog and Oct4, jointly or individually and, most importantly, looking at the functional categories associated with these genes (gene-ontology analysis). To do this, we fused genomic gene expression profiling data from loss of function (LoF) and gain of function (GoF) experiments of Nanog and Oct4 with genome-wide chromatin-immunoprecipitation (ChIP) data. ChIP data provides evidence on the direct protein-DNA binding interactions, whereas Loss and Gain of Function data gives information on functionality. In particular, in the latter case (LoF and GoF data) we considered those genes that were differentially expressed as possible Nanog or Oct4 targets irrespective if their expression is increased or reduced with respect to the control. In this manner, we aimed at finding those genes directly regulated (activated or repressed) by Oct4 or Nanog.
Table SIII summarizes the data sets included in the reanalysis.

Experiment	Publication	Description
**Oct4 and Nanog Gain of Function**	(Nishiyama et al, 2009)	**Conditions:** MC1 cells with Tet-inducible Oct4 or Nanog transgene (knocked-in for ROSA-TET) in S+L conditions. Doxycycline removed 48h before harvesting.<br>**Platform:** Agilent Mouse 44k mircorarray<br>**Data source:** NIA Array Analysis http://lgsun.grc.nia.nih.gov/ANOVA/<br>**Statistical criterion:** Fold-Change greater than 1.5.
**Nanog Loss of Function**	(Loh et al, 2006)	**Conditions:** E14 cells in S+L transfected with a plasmid expressing a Nanog siRNA sequence.<br>**Platform:** Affymetrix Mouse Genome 430 2.0 microarray<br>**Data source:** Loh et al. 2006 SOM<br>**Statistical criterion:** Same Significance Analysis of Microarray (SAM) as in Loh et al. 2006, with median FDR lower than 0.001.
**Oct4 Loss of Function**	(Sharov et al, 2008)	**Conditions:** ZHBTc4 cells in S+Lwith Tetracycline. Harvested at 0, 3, 6, 12, 24, 48, 72, 96 and 108 hours.<br>**Platform:** Agilent Mouse 44k mircorarray<br>**Data source:** NIA Array Analysis http://lgsun.grc.nia.nih.gov/ANOVA/<br>**Statistical criterion**: Genes with a Fold-Change of the response magnitude (max. FC of the time-course) greater than 1.5, and FDR<0.05.
**Nanog and Oct4 binding to DNA**	(Chen et al, 2008)	**Conditions:** E14 cells in S+L. Chromatin Immunoprecipitation against Oct4/Nanog coupled with massively parallel short-tag-based sequencing (ChIP-seq) was used to map DNA binding locations.<br>**Data source:** Chen et al. 2009 SOM<br>**Statistical criterion:** Genes with a positive TF-Gene Association Score (Chen et al. 2009).

Table SIII: Data sets included in the Bioinformatics analysis

As the experiments were performed in different platforms, the first step in our analysis was to identify genes that were tested in all experiments. Using the Mouse Genome Informatics (MGI) Marker Accession IDs or, alternatively, their Official Gene Symbol, we matched 17927 genes that were then tested for expression changes in both Nanog/Oct4 LoF and GoF microarray experiments. We applied specific statistical criteria to this list of genes (background) based on filtering by fold-change and thresholding p-values with the false-discovery rate method, to identify differentially expressed genes (see Table SIII for details of specific statistical criteria used in each data set). In addition, we used ChIP-Seq data ((Chen et al, 2008) Table SIII) to tag genes as being bound by Nanog and/or Oct4. Figures S9A,B show the number of genes detected with each method.

We consider a gene to be an Oct4 (Nanog) target if it is differentially expressed in either LoF or GoF experiments and, in addition, if there is evidence that Oct4 (Nanog) binds close to its transcription start, as established by a positive TF-Gene Association Score computed in (Chen

et al, 2008). Using this criterion we found 2716 Oct4 and 1328 Nanog target genes (see Fig. S9A,B and Supplementary File 1) with a significant overlap of target genes (438 common targets, Fisher's exact test p-value$<10^{-15}$, see Fig 3A), which we deem to be jointly regulated, but also a large amount of genes that are specific targets of either Nanog (890, 67%) or Oct4 (2278, 83%) (Fig. 3 in main text).

Given the significant but incomplete overlap of Nanog and Oct4 target genes detected, we wanted to explore if each of the three groups identified is associated with a particular biological function. To that end we used gene annotations from MGI (www.informatics.jax.org) and performed a standard Gene Ontology (GO) analysis with p-values of Biological Process GO terms assessed by Fisher's exact test. Figure S3B in main text shows the general Biological Process terms that are more strongly overrepresented by Oct4 and Nanog common and specific target genes, with its associated significance value (measured by $\log_{10} p$, where p is the corresponding p-value). This figure shows that, while Cellular and Metabolic processes are highly overrepresented by Oct4 targets, Developmental processes are at least as well represented amidst the Oct4 targets as they are amidst the Nanog targets (756 of 2716 genes, p-value$=1.1\cdot10^{-15}$ for Oct4 while for Nanog 391 of 1328 genes, p-value$=2.2\cdot10^{-11}$). This analysis indicates that while both factors target a large number of genes involved in developmental processes, Oct4 also has a very large number of targets associated with basic biochemical and cellular events. The significance of this targeting will have to be addressed experimentally. One other feature of our analysis is that neither Nanog nor Oct4 targets are associated neither with late differentiation events nor with specialized activities of differentiated cells.

Given the large number of GO terms overrepresented by, in particular, the list of Oct4 target genes, we focused our further analysis only on those terms of particular interest in pluripotency and early lineage differentiation. Specifically, we analysed GO terms that in their description contained at least one of the following words: "development", "differentiation", "ectoderm", "embryo", "endoderm", "epithelial", "mesoderm", "morphogenesis", "neural", "organ", "stem" and "tissue". We further filtered the search to those GO terms with at least 100 genes annotated, a depth on the GO graph of at most 3 (this is, either their parents or the parents of their parents are the "Biological Process" root term) and that at least one of the p-values of Nanog or Oct4 target genes was below 10^{-6}. This procedure yielded a meaningful and hierarchical list of terms, which is shown in Fig. S9C, and that reveals that the set of Oct4 targets is, in part, made up of many

development/differentiation-related genes, which, unlike Nanog targets, are not involved in very specific differentiation processes.

To conclude the comparison of Oct4 and Nanog targets, we tried to identify those genes with transcription factor (TF) and signalling (SGN) activity. For this purpose, we rely on the GO Molecular Function annotation from MGI (term GO:0003700: "sequence-specific DNA binding transcription factor activity") or the Biological Process annotation (term GO:0023052: "signalling"). We were able to identify 175 Oct4 target genes and 86 Nanog target genes with TF activity, of which 40 are common targets. In the case of SGN activity, we recognised 669 Oct4 and 342 Nanog targets involved in signalling activity, 126 of which were shared (TF and SGN activity annotated in Supplementary File 1). Both TF and SGN activity was significantly overrepresented in Nanog and Oct4 target genes (Fisher exact test p-values $<2\cdot10^{-10}$ for TF in Oct4, $<2\cdot10^{-5}$ for TFs in Nanog, $<2\cdot10^{-9}$ for SGN in Oct4 , $<5\cdot10^{-7}$ for SGN in Nanog). Inspection of the list of TFs (Figs. 3A and S9E, right, and Supplementary File 1) reveals striking functional differences between the three groups: the jointly regulated TFs include elements of the core of the pluripotency network (Esrrb, Nanog, Oct4, Klf4, 6, Otx2), while targets of Nanog, and more clearly Oct4, include genes involved in lineage specification (members of the En, Fox, Gata, Hox, T-box, Zic families). The list of signalling molecules (Fig. S9E, left, and Supplementary File 1) also highlights the differences between the different targets: joint targets are not significantly enriched in any particular signalling class, though we notice the presence of Fgf4 and Fgfr2 in this list, both of which have been implicated in fate decisions in the early embryo (Guo et al, 2010). When looking at the list of specific targets of Nanog and Oct4, we observe an interesting symmetry with several Fgf genes as targets of Nanog, and many Fgf receptors and elements of Ras/ERK signalling as targets of Oct4, also including a number of Wnt genes (see Fig. S9E, left, and Supplementary File 1).

Taken together, the analysis of GO terms and the identified TFs and SGN component targets, support the notion that an excess of Oct4 over Nanog will lead to the activation or repression of genes involved in differentiation.

Simulation and results of the behavior of the network in different growth conditions and in mutants for its components elements.

Figures S10-S16 display the results of simulations of the TBON model under different signalling conditions. This is theoretically achieved by adapting the parameter values of the equations and adding/removing reactions to the system based on known evidence and

experimental observations. Changes introduced in the model to account for these perturbations are summarized below, based on experimental observations.

Nanog mutant cells (Figs. S10-S11)

In the absence of Nanog, cells exhibit a high rate of differentiation, as shown in Alkaline phosphatase colony-forming assays (Fig. S10). In this experiment the behavior of Nanog mutant ES cells (44CRE6) was compared to that of their parental heterozygotes (CKO44); for details see (Chambers et al, 2007). The results show the outcome of assays in which 600 ES cells were plated at clonal density in Serum and LIF. After 12 hours the medium was changed to that indicated in the different conditions, and after five days the growth was stopped and Alkaline Phosphatase (a marker of pluripotency) was applied. Colonies were classified into four classes according to their structure and intensity of the stain (see right hand side of the figure for the different classes): white and loose colonies indicate differentiation, tight, pink and loose colonies represent undifferentiated cells; mixed colonies include differentiating (white) and undifferentiated (pink) cells; finally, compact ball-shaped dark-pink colonies ('2i-like') were considered to be composed of cells in the ground state. Nanog mutant cells exhibit low clonality and high differentiation rate, which is rescued in 2i+LIF. Analysis of the components shows that this effect is largely due to Chiron, which acts on β-catenin.

Figure S11A-D shows simulations of the behavior of Nanog null ($Nanog^{-/-}$) cells in S+L. In these simulations, the activity of both Nanog promoters is eliminated, while all other parameter values are taken from S+L. For simulations in 2i+L conditions (Figs. S11E-H), parameter values are taken from 2i+L. Notice that the TBON model is able to predict the observed increased Oct4 and β-catenin levels in both culture conditions (Fig. S11B,C,F,G and 4C) and predicts an increased number of free Oct4 molecules that is reduced upon stabilization of β-catenin (Fig. S11A,E). In the TBON we consider that PD03 acts promoting Nanog expression; in the absence of Nanog, this means that PD03 would have little effect as we observe experimentally (Figs. 4A and S10).

β-catenin mutant cells (Fig. S12-13)

Experimentally, we compared the results of the $\beta\text{-catenin}^{-/-}$ cells with the $\beta\text{-catenin}^{+/-}$ cells that were used as parental line to derive the mutants. The cells were grown in 2i+L conditions ((Faunes et al, 2013; Wray et al, 2011) Fig. S12A-D). For the simulations we assume the following: in the heterozygous cells the supply of β-catenin is approximately halved ($\mu_{\beta+} = 150$ and $\sigma^2_{\beta+} = 0.1$), and all other parameter values are taken from 2i+L; in the mutant cells, β-catenin supply is completely removed ($k_{\beta+} = 0$), and all other parameter values are taken from 2i+L (Fig. S12E-L).

When β-catenin mutant cells are cultured in S+L, they have a high tendency to differentiate (Faunes et al, 2013; Wray et al, 2011). For this reason, we wanted to know what the TBON model would predict about the inner state of the cells under these conditions (Fig. S13). As in the 2i+L simulation, we considered that β-catenin supply is approximately halved in β-catenin heterozygotes, ($\mu_{\beta+} = 150$ and $\sigma^2_{\beta+} = 0.1$), and all other parameter values are taken from S+L. For β-catenin null cells, β-catenin supply is completely removed ($k_{\beta+} = 0$), and all other parameter values are taken from S+L. In this situation, the model predicts an increase in the free Oct4 and Tcf3 levels that could be responsible for the differentiation of these cells (Fig. S13F, and compare panels G with H).

Tcf3 mutant cells (Fig. S14)

To simulate Tcf3 mutant cells, the two Tcf3 promoters are removed from the system, while all other parameter values are taken from S+L. We additionally included observed experimental effects, in particular the reduction of Oct4 and β-catenin production ($k_{+3} = 0.2, \mu_{\beta+} = 150$). One striking feature of the Tcf3 mutants is the existence a stable population of cells in the lower O/N ratio boundary, a population that does not normally exist due to the instability of free Nanog (Fig. S14F). This suggests a scenario in which the absence of Tcf3 allows an increased stability of Nanog, therefore in our simulations we increased the stability of free Nanog ($k_{14} = 0.8$) (Figs. 5C,D and S14G-N). All other parameter values are taken from S+L.

For simplicity, the $Tcf3^{+/+}$ parental cells are assumed to be identical (same parameter values) to the wild type Tg2A cells in S+L.

Oct4 loss of function (Fig. S15)

ZHBTc4 cells are Oct4 heterozygous cells in which the functional allele has been modified so that it is inactive and the other one is under doxycycline control (Niwa et al, 2002). Addition of doxycycline to a culture of these cells results in a rapid decrease in the levels of Oct4 (Fig. S15A-C). For the simulations we consider only one Oct4 promoter. Upon start of doxycycline treatment, Oct4 transcription is halted ($k_6 = 0$), while all other parameter values are taken from S+L. The inability of maintaining the pluripotent state in these cells comes from the fact the Nanog levels constantly decrease as Oct4 disappears from the system. The decrease of Oct4 that we observe experimentally is well reproduced by the model simulation (compare panels B,C with E,F from Fig. S15).

Oct4 overexpression (Fig. S16)

AG23191 cells overexpress Oct4 from a TetOFF system, so that removal of Dox from the culture leads to overexpression of Oct4 (Nishiyama et al, 2009). We simulate the behaviour of the TBON in these cells by considering an additional gene of Oct4 with continuous expression ($P_{tet} \xrightarrow{k_O} P_{tet} + mO$) with $k_O = 5$, while all other parameter values are taken from S+L or Chiron conditions (see main text). Extra production of Oct4 in S+L conditions leads to stabilization of Nanog (Fig. S16C), which is not able to mop up all excess free Oct4 (Figs. S16F and 6Din main text). Extra β-catenin supply to the system via Chiron compensates the excess of Oct4 and reduces the levels of free protein to its normal condition (Figs. S16F and 6D).

Simulation of the decay of the TBON elements after cyclohexamide treatment

Cyclohexamide treatment was used experimentally (Fig. S3) to estimate protein half-lives. In order to model cyclohexamide treatment for wild-type cells in S+L (Fig. S17) and compare it with the experimental results shown in Fig. S3, we consider that protein translation is halted ($k_8, k_9, k_{10}, k_{+\beta} = 0$), and all other parameter values are taken from S+L. Notice that the simulations reproduce the distributions obtained experimentally.

Effect of a self-repressive feedback loop on Nanog

Recent experimental work points to the existence of a negative feedback loop of Nanog onto itself (Navarro et al, 2012). In order to investigate the effect of such feedback in the pluripotency network, we introduced new reactions in the TBON model to account for this regulatory step (Fig. S19A):

$$P_{N,0}^{\alpha} + N \xrightarrow{k_{+N}} P_{N,3}^{\alpha} \qquad P_{N,3}^{\alpha} \xrightarrow{k_{-N}} P_{N,0}^{\alpha} + N \qquad \alpha = 1,2.$$

These reactions describe the inhibition of the Nanog promoter by the binding to DNA of Nanog proteins. The strength of the feedback is controlled by rates of binding and unbinding (k_{+N}, k_{-N}). We have investigated the case where the effect of the negative feedback is moderate ($k_{+N} = 0.005, k_{-N} = 2$).

Both in S+L and 2i+L conditions the effect of the negative feedback in the protein distributions and correlations is not altered (Fig. S18B-D). Figure S18E shows that in S+L, the active state (ON) in the Nanog promoters is not substantially altered by the presence of an autorepressive feedback, as the promoter is already in a highly repressed state. In 2i+L (Fig. S18F), however, the continuous production of Nanog mRNA is markedly reduced by the presence of Nanog autorepression.

<u>Supplementary Figure legends:</u>

Figure S1: A transcriptional circuit fails to reproduce the protein correlation data. (A) Scheme of the core network maintaining pluripotency. In the figure, solid lines represent direct interactions and dashed lines depict indirect connections. **(B)** Simplified transcriptional regulatory network. **(C)** Behaviour of the system considering the network shown in B. The blue and green lines are nullclines of the deterministic equations. Each dot represents the state of a single cell obtained by stochastic simulation of the transcriptional model. For details see text.

Figure S2: Effect of proteasome inhibition on Nanog, Oct4 and β-catenin. (A) Representative confocal images of E14Tg2A cells stained for Dapi (blue), Nanog (green), β-catenin (red) and Oct4 (white) grown in S+L (upper panels) or treated with MG132 for 2h (lower panels). Scale bar: 50 μm. **(B-D)** Protein distributions of Nanog (B), Oct4 (C) and β-catenin (D) in E14Tg2A cells in S+L (S+L) or treated with MG132 for 2 h (S+L MG 2h) indicate that 2h of proteasome inhibition lead to an increase in the levels protein levels and a reduction of the heterogeneity, as shown by the Coefficient of Variation (CV). Fluorescence levels (grayscale) were quantified for each individual cell from four colonies, binned in logarithmically spaced classes (x-axis); the frequency of each bin is shown on the y-axis. Experimental protein distributions in similar subsequent graphs were obtained using the same method.

Figure S3: Measurements of Oct4, Nanog, Tcf3 and β-catenin half-lives. (A) *β-catenin, Tcf3, Nanog* and *Oct4* mRNA half-lives (from (Sharova et al, 2009)). **(B)** Western Blot showing β-catenin, Tcf3, Nanog and Oct4 levels after treating mES cells for 0h (control), 2h, 4h, and 8h with cyclohexamide (CHX). Tubulin is a loading control. **(C-F)** Scatter plots showing Nanog (x-axis), Oct4 (y-axis) and β-catenin (heat-map) levels in fluorescence arbitrary units (au; here and in subsequent similar graphs) in single E14Tg2A cells under standard S+L treated with DMSO (control, C) or CHX for 2h (D), 4h (E), or 8h (F). Each dot represents the levels in one single cell. **(G-J)** Comparison of WB and QIF results. Average levels of proteins normalized to levels at 0h. Dark colours show the results from the WB experiments, while lighter ones correspond to QIF measurements. Panel G shows the results for Nanog; H, for Oct4; I, for β-catenin; and J, for Tcf3. Half-life was determined by fitting a first order decay model in both WB data (grey line) and QIF data (not shown). **(K)** Comparison of the half-lives obtained results from WB and QIF. Notice that the 8h point in

QIF is higher than expected for β-catenin and Tcf3, and this might introduce a large extrapolation error in the half-life estimate. In these cases we simply consider that the half-lives are on the order of 10h (asterisk).

Figure S4: A minimal competitive protein interaction network: The NOC model. (A-B) Time traces of Nanog (blue) and Oct4 (green) promoter activity (upper panels), mRNA (middle panels) and protein (lower panels) levels in cells grown in S+L (A) or 2i+L (B) conditions. For simplicity, here and in subsequent figures, we include a light vanilla background when showing modelling results. **(C)** Simulated Nanog mRNA molecule distributions in cells grown in S+L (light blue line) and 2i+L (dark blue line). **(D)** Simulated Oct4 mRNA molecule distributions in cells grown in S+L (light blue line) and 2i+L (dark blue line). **(E)** Simulated Nanog protein distributions in cells grown in S+L (light blue line) and 2i+L (dark blue line). **(F)** Simulated Oct4 protein distributions in cells grown in S+L (light blue line) and 2i+L (dark blue line). **(G)** Sample trajectories simulated with the deterministic NOC model are depicted in the (N,O,O:N) state space, to show the effect of fast Nanog decay. Each trajectory has been given initial pulses of Nanog and Oct4 production of different amplitudes. **(H)** The same trajectories in (G) represented in the (N_t,O_t) space reveal the boundary at the O/N=1 ratio. **(I)** Nanog promoter dynamics is a key factor controlling the final O/N correlation. Variation of the promoter timescale ϕ does not affect the ratio of promoter activity (top) nor the average number of mRNA molecules (center) but modifies the O/N correlation (bottom panel). **(J-L)** Effect of the promoter timescale in the O-N distribution for 3 different cases.

Figure S5: Generalisation of the NOC model to multimeric interactions. (A-B) Simulated scatter plots showing Nanog (x-axis) and Oct4 (y-axis) number of molecules under standard S+L (black dots) and 2i+L (red dots) conditions, considering that a Nanog dimer binds one Oct4 molecule (A) or an Oct4 dimer (B). Each dot represents one single cell. **(C)** Pearson correlation coefficient between Oct4 and Nanog (O/N) in cells cultured in S+L and 2i+L for the NOC model (black bars) and for the multimeric variants shown in panels (A) and (B) (grey and white bars respectively.).

Figure S6: Details on the TBON model. (A-D) Protein distributions of Nanog (A), Oct4 (B), β-catenin (C) and Tcf3 (D) in E14Tg2A cells grown in S+L (S+L, light colour lines) or in 2i+L conditions (dark colour lines), obtained as in Fig. S3. **(E-H)** Simulated protein distributions of Nanog (E), Oct4 (F), β-catenin (G) and Tcf3 (H) in E14Tg2A cells grown in S+L (S+L, light colour lines) and in 2i+L conditions (dark colour lines). **(I)** Simulated Nanog

mRNA molecule distributions in cells grown in S+L (light blue line) and 2i+L (dark blue line). **(J)** Simulated Oct4 mRNA molecule distributions in cells grown in S+L (light blue line) and 2i+L (dark blue line). **(K)** Average fraction of time Nanog promoters in each of the possible states: ON (active), OFF (inactive) and IN (inhibited) under the indicated culture conditions. **(L)** Simulated free Oct4 protein distributions in E14Tg2A cells grown in S+L (S+L, light colour lines) and in 2i+L conditions (dark colour lines). **(M)** Complementary cumulative distributions of free Oct4 molecules in the indicated growing conditions.

Figure S7: Robustness of the correlations of the TBON model to parameter perturbations. A parameter sensitivity analysis of the TBON model indicates that the O/N (A), B/N (B) and B/O (C) correlations are not strongly affected by a 25% increase (maroon bars) or decrease (blue bars) in the post-translational parameter values. Vertical red lines indicating the Pearson correlation values in S+L simulated conditions are shown for comparison.

Figure S8: Effects of Chiron and PD03 on the activity of the network. (A-B) Simulated scatter plots showing Nanog (x-axis), Oct4 (y-axis) and β-catenin (heat map) levels in single E14Tg2A cells under Chiron treatment (A) or PD03 (B). **(C)** Simulated free Oct4 protein distributions in E14Tg2A cells in the indicated conditions. **(D)** Complementary cumulative distribution of free Oct4 molecules in the indicated growing conditions. **(E-H)** Simulated protein distributions of Nanog (E), Oct4 (F), β-catenin (G) and Tcf3 (H) in E14Tg2A cells grown in Chiron (light colour lines) or PD03 (dark colour lines). **(K)** Average fraction of time Nanog promoters in each of the possible states: ON (active), OFF (inactive) and IN (inhibited) under Chiron or PD03 treatment. **(J-K)** Pie charts showing the relative pools of Nanog, Oct4, β-catenin and Tcf3 found as free molecules (dark colours) or as part of a complex (pale colours), as determined by the TBON model in cells treated with Chiron (I) or PD03 (J).

Figure S9: TBON – Analysis of Oct4 and Nanog target genes. (A) Venn diagrams showing the sets of genes identified as differentially expressed in Oct4 Loss-of-Function (LoF) and Gain-of-Function (GoF) studies, and also genes for which there is evidence of Oct4 binding close to their DNA transcription start region. (**B**) Corresponding Venn diagrams for Nanog. (**C**) Nanog and Oct4 target genes mapped to their "biological process" gene ontologies. Only general categories are shown. **(D)** Network diagram showing the common and specific target genes of Nanog and Oct4 identified by our bioinformatics analysis. Red nodes indicate target genes with known transcription factor activity, green nodes denote genes with signalling

activity, and yellow indicate that both types of activity have been previously identified. **(E)** List of selected target genes, classified according to their transcriptional or signalling activity.

Figure S10: Stability of pluripotency in Nanog mutant cells. Colony forming assays for $Nanog^{+/+}$ and $Nanog^{-/-}$cells under the indicated conditions. Colonies were assayed for alkaline phosphatase (AP, a marker for pluripotency) and scored as differentiated, AP negative or weakly positive, and undifferentiated, AP strongly positive. For details of classification, see text. **(A)** Absolute number of the different colony types from the relative percentages shown in Fig. 4A. **(B)** Relative percentages and **(C)** absolute numbers of the different colony types considering the addition of LIF to the cultured conditions. The micrographs show the different types of colonies observed.
See SM text for details.

Figure S11: Simulations of Nanog mutant cells in S+L and 2i+L. (A-D) Simulations of Nanog wild type and null cells grown in S+L conditions. **(E-H)** Simulations of Nanog null cells grown in 2i+L conditions. **(A, E)** Simulated free Oct4 protein distributions. **(B, F)** Simulated total Oct4 protein distributions. **(C, G)** Simulated total β-catenin protein distributions. **(D, H)** Simulated total Tcf3 protein distributions.

Figure S12:Simulations of β-catenin mutant cells grown in 2i+L. (A-D) Experimental results of β-$catenin^{+/-}$ and β-$catenin^{-/-}$ cells grown in 2i+L conditions. **(E-J)** Simulations of β-$catenin^{+/-}$ and β-$catenin^{-/-}$ cells grown in 2i+L conditions. **(A,E)** Nanog protein distributions. **(B,F)** Total Oct4 protein distributions. **(C,D,G,H)** Scatter plots showing Nanog (x-axis) and Oct4 (y-axis) single β-$catenin^{+/-}$ (C,G) and β-$catenin^{-/-}$ cells (D,H). **(I)** Simulated free Oct4 molecule distribution. **(J)** Simulated free Tcf3 molecule distribution. **(K)** Pearson correlation coefficient between Oct4 and Nanog from the experimental data (grey bars), and in the simulations (black bars), in wild-type, β-$catenin^{+/-}$ and β-$catenin^{-/-}$ cells cultured in 2i+L. **(L)** Pie charts showing the relative pools of Nanog, Oct4, β-catenin and Tcf3 found as free molecules (dark colours) or as part of a complex (pale colours), as determined by the TBON model in β-$catenin^{+/-}$ cells grown in 2i+L.

Figure S13: Simulations of β-catenin mutant cells grown in S+L. Simulations of β-catenin cells grown in S+L conditions. **(A-B)** Scatter plots showing Nanog (x-axis) and Oct4 (y-axis) single β-$catenin^{+/-}$ (A) and β-$catenin^{-/-}$ cells (B). **(C-E)** Total protein distributions of Nanog (C), Oct4 (D), and Tcf3 (E). **(F)** Simulated free Oct4 molecule distribution. **(G-H)** Pie charts showing the relative pools of Nanog, Oct4, β-catenin and Tcf3 found as free molecules (dark

colours) or as part of a complex (pale colours), as determined by the TBON model in β-catenin$^{+/-}$ (G) and β-catenin$^{-/-}$ (H).

Figure S14: Tcf3 mutant cells. **(A)** Representative confocal images of Tcf3$^{+/+}$ (parental line, upper panels) and Tcf3$^{-/-}$ (lower panels) cells stained for Nanog (green), Oct4 (red), and total β-catenin (white) grown in S+L. Scale bar: 50 µm. **(B-F)** Experimental results of Tcf3$^{+/+}$ and Tcf3$^{-/-}$ cells. **(G-N)** TBON simulations of Tcf3 mutant cells**.** **(G)** Simulated total Nanog protein distribution. **(B)** Simulated total Oct4 protein distribution. **(C)** Simulated total β-catenin protein distribution. **(D)** Scatter plot showing Nanog (x-axis), Oct4 (y-axis) and β-catenin (heat-map) in single Tcf3$^{-/-}$ cells. **(E)** Simulated free Oct4 molecule distribution. **(F)** Complementary cumulative distribution of free Oct4 molecules. **(G)** Nanog promoter activity in Tcf3$^{+/+}$ and Tcf3$^{-/-}$ cells.

Figure S15: Effect of Oct4 loss of function. Oct4 was depleted using ZHBTc4 cells, which contain a tetracycline-inducible Oct4 deletion. **(A)** Representative confocal images of ZHBTc4 cells cultured in S+L without DOX (upper panels) and with DOX for 24h (middle panels) and 48h (lower panels) stained for Tcf3 (green), Nanog (red), Oct4 (magenta) and total β-catenin (white). Scale bar: 50 µm. **(B-D)** Experimental results. **(E-G)** Simulations. **(B,E)** Scatter plots showing Nanog (x-axis) and Oct4 (y-axis) levels in single cells cultured in S+L (blue dots), or treated with DOX for 24h (green dots) or 48h (red dots). **(C,F)** Total Oct4 protein distributions. **(D, G)** Total β-catenin protein distributions.

Figure S16: Effect of Oct4 overexpression. Oct4 was overexpressed using AG-23191 cells, which contain a Dox-inducible Oct4 transgene (Nishiyama et al, 2009). **(A-B)** Experimental protein distributions of Oct4 (A) and β-catenin (B) in the indicated growing conditions. **(C-E)** Simulated protein distributions of total Oct4 (C), total β-catenin (D) and total Tcf3 (E). **(F)** Simulated free Oct4 distribution. **(G-J)** Experimental scatterplots showing Nanog (x-axis), Oct4 (y-axis) and β-catenin (heat-map) in single AG-23191 in the indicated growing conditions. **(K-L)** Simulated scatterplots upon Oct4 overexpression (K) and Oct4 overexpression and Chiron treatment (L).

Figure S17: Simulation of the decay of the TBON elements after cyclohexamide treatment. **(A-D)** Simulated scatterplots showing Nanog (x-axis), Oct4 (y-axis) and β-catenin (heat-map) in single Tg2A cells in S+L (A) or after stopping protein synthesis with cyclohexamide (CHX) for 2h (B), 4h (C) or 8h (D). Compare with the experimental results shown in Fig. S2C-F. **(E-F)** Simulated average protein levels after stopping protein synthesis,

normalized to time 0h. Interquartile range is shown as coloured bands as a measure of the evolution of the variance of the protein distributions. **I)** Nanog, Oct4, Tcf3 and β-catenin half-lives estimated using TBON.

Figure S18: TBON – Nanog self-repressive feedback loop. (A) Scheme of the core protein TBON network as shown in Fig. 2B, including a Nanog self-repressive feedback loop. **(B-C)** Simulated scatterplots showing Nanog (x-axis), Oct4 (y-axis) and β-catenin (heat map) considering a negative feedback loop in the indicated culture conditions. **(D)** Pearson correlation coefficient between Oct4 and Nanog (O/N), β-catenin and Nanog (β/N) and β-catenin and Oct4 (β/O) from the experimental data (grey bars), and in the TBON simulations considering the feedback loop (black bars) in cells cultured in S+L and 2i+L.
(E-F) Nanog promoter activity considering the repressive feedback loop in cells cultured in S+L (E) or 2i+L (F). Here PT and PN indicate Nanog promoter repression by Tcf3 and Nanog respectively.

References

Abranches E, Bekman E, Henrique D (2013) Generation and characterization of a novel mouse embryonic stem cell line with a dynamic reporter of Nanog expression. *PLoS One* **8:** e59928

Abu-Remaileh M, Gerson A, Farago M, Nathan G, Alkalay I, Zins Rousso S, Gur M, Fainsod A, Bergman Y (2010) Oct-3/4 regulates stem cell identity and cell fate decisions by modulating Wnt/beta-catenin signalling. *EMBO J* **29:** 3236-3248

Alon U (2007) Network motifs: theory and experimental approaches. *Nat Rev Genet* **8:** 450-461

Becskei A, Serrano L (2000) Engineering stability in gene networks by autoregulation. *Nature* **405:** 590-593

Campbell PA, Perez-Iratxeta C, Andrade-Navarro MA, Rudnicki MA (2007) Oct4 targets regulatory nodes to modulate stem cell function. *PLoS One* **2:** e553

Carey BW, Markoulaki S, Hanna JH, Faddah DA, Buganim Y, Kim J, Ganz K, Steine EJ, Cassady JP, Creyghton MP, Welstead GG, Gao Q, Jaenisch R (2011) Reprogramming factor stoichiometry influences the epigenetic state and biological properties of induced pluripotent stem cells. *Cell Stem Cell* **9:** 588-598

Chambers I, Silva J, Colby D, Nichols J, Nijmeijer B, Robertson M, Vrana J, Jones K, Grotewold L, Smith A (2007) Nanog safeguards pluripotency and mediates germline development. *Nature* **450:** 1230-1234

Chambers I, Tomlinson SR (2009) The transcriptional foundation of pluripotency. *Development* **136:** 2311-2322

Chen X, Xu H, Yuan P, Fang F, Huss M, Vega VB, Wong E, Orlov YL, Zhang W, Jiang J, Loh YH, Yeo HC, Yeo ZX, Narang V, Govindarajan KR, Leong B, Shahab A, Ruan Y, Bourque G, Sung WK et al (2008) Integration of external signaling pathways with the core transcriptional network in embryonic stem cells. *Cell* **133:** 1106-1117

Chew JL, Loh YH, Zhang W, Chen X, Tam WL, Yeap LS, Li P, Ang YS, Lim B, Robson P, Ng HH (2005) Reciprocal transcriptional regulation of Pou5f1 and Sox2 via the Oct4/Sox2 complex in embryonic stem cells. *Mol Cell Biol* **25:** 6031-6046

Ding J, Xu H, Faiola F, Ma'ayan A, Wang J (2012) Oct4 links multiple epigenetic pathways to the pluripotency network. *Cell Res* **22:** 155-167

Faunes F, Hayward P, Muñoz-Descalzo S, Chatterjee SS, Balayo T, Trott J, Christophorou A, Ferrer-Vaquer A, Hadjantonakis AK, DasGupta R, Martinez Arias A (2013) A membrane-associated β-catenin/Oct4 complex correlates with ground-state pluripotency in mouse embryonic stem cells. *Development* **140:** 1171-1183

Festuccia N, Osorno R, Halbritter F, Karwacki-Neisius V, Navarro P, Colby D, Wong F, Yates A, Tomlinson SR, Chambers I (2012) Esrrb is a direct Nanog target gene that can substitute for Nanog function in pluripotent cells. *Cell Stem Cell* **11:** 477-490

Fidalgo M, Faiola F, Pereira CF, Ding J, Saunders A, Gingold J, Schaniel C, Lemischka IR, Silva JC, Wang J (2012) Zfp281 mediates Nanog autorepression through recruitment of the NuRD complex and inhibits somatic cell reprogramming. *Proc Natl Acad Sci U S A* **109:** 16202-16207

Guo G, Huss M, Tong GQ, Wang C, Li Sun L, Clarke ND, Robson P (2010) Resolution of cell fate decisions revealed by single-cell gene expression analysis from zygote to blastocyst. *Dev Cell* **18:** 675-685

Hayashi K, Lopes SM, Tang F, Surani MA (2008) Dynamic equilibrium and heterogeneity of mouse pluripotent stem cells with distinct functional and epigenetic states. *Cell Stem Cell* **3:** 391-401

Kalmar T, Lim C, Hayward P, Munoz-Descalzo S, Nichols J, Garcia-Ojalvo J, Martinez Arias A (2009) Regulated fluctuations in nanog expression mediate cell fate decisions in embryonic stem cells. *PLoS Biol* **7:** e1000149

Kelly KF, Ng DY, Jayakumaran G, Wood GA, Koide H, Doble BW (2011) beta-catenin enhances Oct-4 activity and reinforces pluripotency through a TCF-independent mechanism. *Cell Stem Cell* **8:** 214-227

Kim J, Chu J, Shen X, Wang J, Orkin SH (2008) An extended transcriptional network for pluripotency of embryonic stem cells. *Cell* **132:** 1049-1061

Lanner F, Lee KL, Sohl M, Holmborn K, Yang H, Wilbertz J, Poellinger L, Rossant J, Farnebo F (2010) Heparan sulfation-dependent fibroblast growth factor signaling maintains embryonic stem cells primed for differentiation in a heterogeneous state. *Stem Cells* **28:** 191-200

Liang J, Wan M, Zhang Y, Gu P, Xin H, Jung SY, Qin J, Wong J, Cooney AJ, Liu D, Songyang Z (2008) Nanog and Oct4 associate with unique transcriptional repression complexes in embryonic stem cells. *Nat Cell Biol* **10:** 731-739

Loh KM, Lim B (2011) A precarious balance: pluripotency factors as lineage specifiers. *Cell Stem Cell* **8:** 363-369

Loh YH, Wu Q, Chew JL, Vega VB, Zhang W, Chen X, Bourque G, George J, Leong B, Liu J, Wong KY, Sung KW, Lee CW, Zhao XD, Chiu KP, Lipovich L, Kuznetsov VA, Robson P, Stanton LW, Wei CL et al (2006) The Oct4 and Nanog transcription network regulates pluripotency in mouse embryonic stem cells. *Nat Genet* **38:** 431-440

Lu R, Markowetz F, Unwin RD, Leek JT, Airoldi EM, MacArthur BD, Lachmann A, Rozov R, Ma'ayan A, Boyer LA, Troyanskaya OG, Whetton AD, Lemischka IR (2009) Systems-level dynamic analyses of fate change in murine embryonic stem cells. *Nature* **462:** 358-362

Luo Y, Lim CL, Nichols J, Martinez-Arias A, Wernisch L (2012) Cell signalling regulates dynamics of Nanog distribution in embryonic stem cell populations. *J R Soc Interface*

MacArthur BD, Sevilla A, Lenz M, Muller FJ, Schuldt BM, Schuppert AA, Ridden SJ, Stumpf PS, Fidalgo M, Ma'ayan A, Wang J, Lemischka IR (2012) Nanog-dependent feedback loops regulate murine embryonic stem cell heterogeneity. *Nat Cell Biol* **14:** 1139-1147

Martello G, Sugimoto T, Diamanti E, Joshi A, Hannah R, Ohtsuka S, Gottgens B, Niwa H, Smith A (2012) Esrrb is a pivotal target of the Gsk3/Tcf3 axis regulating embryonic stem cell self-renewal. *Cell Stem Cell* **11:** 491-504

Masui S, Nakatake Y, Toyooka Y, Shimosato D, Yagi R, Takahashi K, Okochi H, Okuda A, Matoba R, Sharov AA, Ko MS, Niwa H (2007) Pluripotency governed by Sox2 via regulation of Oct3/4 expression in mouse embryonic stem cells. *Nat Cell Biol* **9:** 625-635

Matoba R, Niwa H, Masui S, Ohtsuka S, Carter MG, Sharov AA, Ko MS (2006) Dissecting Oct3/4-regulated gene networks in embryonic stem cells by expression profiling. *PLoS One* **1:** e26

Miyanari Y, Torres-Padilla ME (2012) Control of ground-state pluripotency by allelic regulation of Nanog. *Nature* **483:** 470-473

Mullin NP, Yates A, Rowe AJ, Nijmeijer B, Colby D, Barlow PN, Walkinshaw MD, Chambers I (2008) The pluripotency rheostat Nanog functions as a dimer. *Biochem J* **411:** 227-231

Munoz Descalzo S, Rue P, Garcia-Ojalvo J, Arias AM (2012) Correlations between the levels of oct4 and nanog as a signature for naive pluripotency in mouse embryonic stem cells. *Stem Cells* **30:** 2683-2691

Munsky B, Neuert G, van Oudenaarden A (2012) Using gene expression noise to understand gene regulation. *Science* **336:** 183-187

Navarro P, Festuccia N, Colby D, Gagliardi A, Mullin NP, Zhang W, Karwacki-Neisius V, Osorno R, Kelly D, Robertson M, Chambers I (2012) OCT4/SOX2-independent Nanog autorepression modulates heterogeneous Nanog gene expression in mouse ES cells. *EMBO J* **31:** 4547-4562

Nguyen H, Merrill BJ, Polak L, Nikolova M, Rendl M, Shaver TM, Pasolli HA, Fuchs E (2009) Tcf3 and Tcf4 are essential for long-term homeostasis of skin epithelia. *Nat Genet* **41:** 1068-1075

Nishiyama A, Sharov AA, Piao Y, Amano M, Amano T, Hoang HG, Binder BY, Tapnio R, Bassey U, Malinou JN, Correa-Cerro LS, Yu H, Xin L, Meyers E, Zalzman M, Nakatake Y, Stagg C, Sharova L, Qian Y, Dudekula D et al (2013) Systematic repression of transcription factors reveals limited patterns of gene expression changes in ES cells. *Sci Rep* **3:** 1390

Nishiyama A, Xin L, Sharov AA, Thomas M, Mowrer G, Meyers E, Piao Y, Mehta S, Yee S, Nakatake Y, Stagg C, Sharova L, Correa-Cerro LS, Bassey U, Hoang H, Kim E, Tapnio R, Qian Y, Dudekula D, Zalzman M et al (2009) Uncovering early response of gene regulatory networks in ESCs by systematic induction of transcription factors. *Cell Stem Cell* **5:** 420-433

Niwa H, Masui S, Chambers I, Smith AG, Miyazaki J (2002) Phenotypic complementation establishes requirements for specific POU domain and generic transactivation function of Oct-3/4 in embryonic stem cells. *Mol Cell Biol* **22:** 1526-1536

Pereira L, Yi F, Merrill BJ (2006) Repression of Nanog gene transcription by Tcf3 limits embryonic stem cell self-renewal. *Mol Cell Biol* **26:** 7479-7491

Rodda DJ, Chew JL, Lim LH, Loh YH, Wang B, Ng HH, Robson P (2005) Transcriptional regulation of nanog by OCT4 and SOX2. *J Biol Chem* **280:** 24731-24737

Rue P, Garcia-Ojalvo J (2011) Gene circuit designs for noisy excitable dynamics. *Math Biosci* **231:** 90-97

Savageau MA (1974) Comparison of classical and autogenous systems of regulation in inducible operons. *Nature* **252:** 546-549

Saxe JP, Tomilin A, Scholer HR, Plath K, Huang J (2009) Post-translational regulation of Oct4 transcriptional activity. *PLoS One* **4:** e4467

Sharov AA, Masui S, Sharova LV, Piao Y, Aiba K, Matoba R, Xin L, Niwa H, Ko MS (2008) Identification of Pou5f1, Sox2, and Nanog downstream target genes with statistical confidence by applying a novel algorithm to time course microarray and genome-wide chromatin immunoprecipitation data. *BMC Genomics* **9:** 269

Sharov AA, Nishiyama A, Piao Y, Correa-Cerro LS, Amano T, Thomas M, Mehta S, Ko MS (2011) Responsiveness of genes to manipulation of transcription factors in ES cells is associated with histone modifications and tissue specificity. *BMC Genomics* **12:** 102

Sharova LV, Sharov AA, Nedorezov T, Piao Y, Shaik N, Ko MS (2009) Database for mRNA half-life of 19 977 genes obtained by DNA microarray analysis of pluripotent and differentiating mouse embryonic stem cells. *DNA Res* **16:** 45-58

Shy BR, Wu C, Khramtsova GF, Zhang JF, O.I. O, Goss KH, Merrill BJ (2013) Regulation of Tcf7l1 DNA binding and protein stability as principle mechanisms of Wnt/β-catenin signaling. *Cell Reports* **In press**

Takao Y, Yokota T, Koide H (2007) Beta-catenin up-regulates Nanog expression through interaction with Oct-3/4 in embryonic stem cells. *Biochem Biophys Res Commun* **353:** 699-705

Tam WL, Lim CY, Han J, Zhang J, Ang YS, Ng HH, Yang H, Lim B (2008) T-cell factor 3 regulates embryonic stem cell pluripotency and self-renewal by the transcriptional control of multiple lineage pathways. *Stem Cells* **26:** 2019-2031

Trott J, Hayashi K, Surani A, Babu MM, Martinez-Arias A (2012) Dissecting ensemble networks in ES cell populations reveals micro-heterogeneity underlying pluripotency. *Mol Biosyst* **8:** 744-752

van den Berg DL, Snoek T, Mullin NP, Yates A, Bezstarosti K, Demmers J, Chambers I, Poot RA (2010) An Oct4-centered protein interaction network in embryonic stem cells. *Cell Stem Cell* **6:** 369-381

van den Berg DL, Zhang W, Yates A, Engelen E, Takacs K, Bezstarosti K, Demmers J, Chambers I, Poot RA (2008) Estrogen-related receptor beta interacts with Oct4 to positively regulate Nanog gene expression. *Mol Cell Biol* **28:** 5986-5995

Wang J, Rao S, Chu J, Shen X, Levasseur DN, Theunissen TW, Orkin SH (2006) A protein interaction network for pluripotency of embryonic stem cells. *Nature* **444:** 364-368

Wray J, Kalkan T, Gomez-Lopez S, Eckardt D, Cook A, Kemler R, Smith A (2011) Inhibition of glycogen synthase kinase-3 alleviates Tcf3 repression of the pluripotency network and increases embryonic stem cell resistance to differentiation. *Nat Cell Biol*

Wray J, Kalkan T, Smith AG (2010) The ground state of pluripotency. *Biochem Soc Trans* **38:** 1027-1032

Yi F, Pereira L, Hoffman JA, Shy BR, Yuen CM, Liu DR, Merrill BJ (2011) Opposing effects of Tcf3 and Tcf1 control Wnt stimulation of embryonic stem cell self-renewal. *Nat Cell Biol*

Yi F, Pereira L, Merrill BJ (2008) Tcf3 functions as a steady-state limiter of transcriptional programs of mouse embryonic stem cell self-renewal. *Stem Cells* **26:** 1951-1960

Young RA (2011) Control of the embryonic stem cell state. *Cell* **144:** 940-954

Zhang L, Rayner S, Katoku-Kikyo N, Romanova L, Kikyo N (2007) Successful co-immunoprecipitation of Oct4 and Nanog using cross-linking. *Biochem Biophys Res Commun* **361:** 611-614

Zhang X, Zhang J, Wang T, Esteban MA, Pei D (2008) Esrrb activates Oct4 transcription and sustains self-renewal and pluripotency in embryonic stem cells. *J Biol Chem* **283:** 35825-35833

Supplemental Figures

Fig. S1: A transcriptional circuit fails to reproduce the protein correlation data

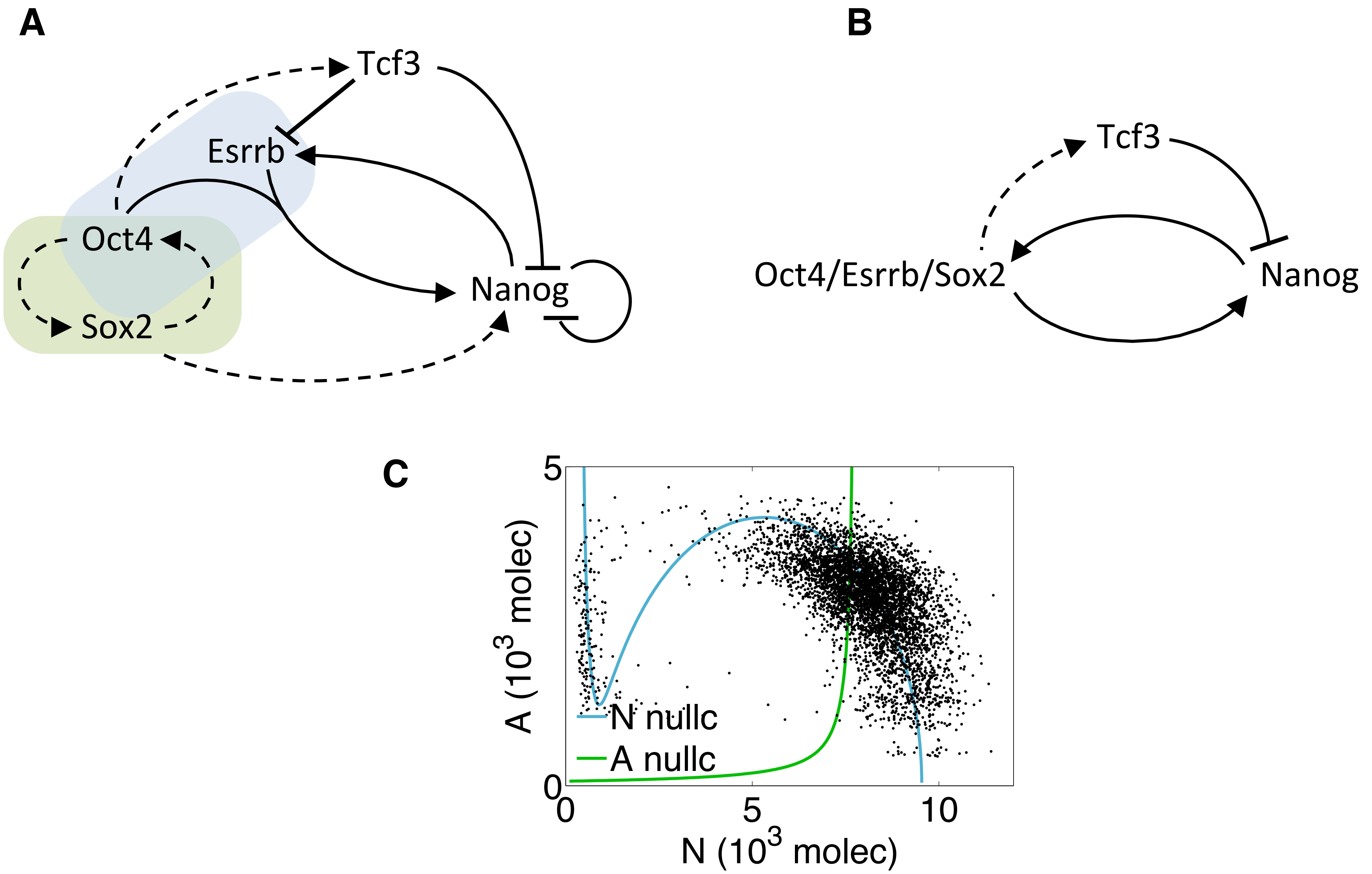

Fig. S2: Effect of proteasome inhibition on Nanog, Oct4 and β-catenin

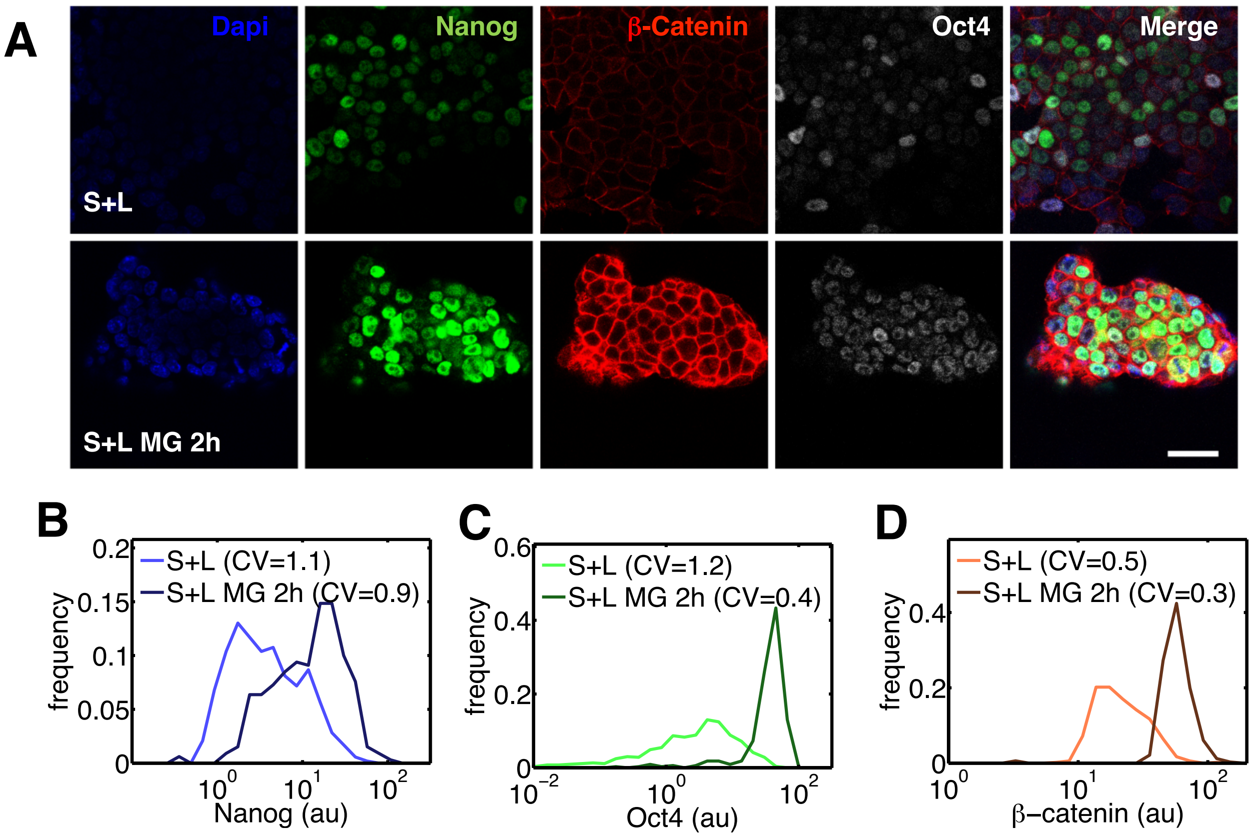

Fig. S3: Measurements of Oct4,Nanog, Tcf3 and β-catenin half-lives

A

Transcript	half life (hours)
Ctnnb1 (β-catenin)	8.8
Tcf3	5.5
Nanog	5.6
Pou5f1 (oct4)	4.6

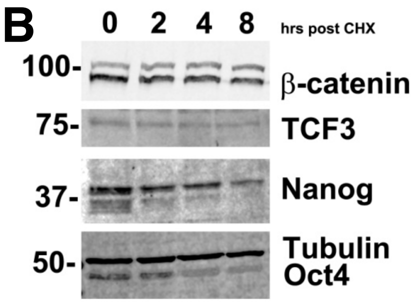

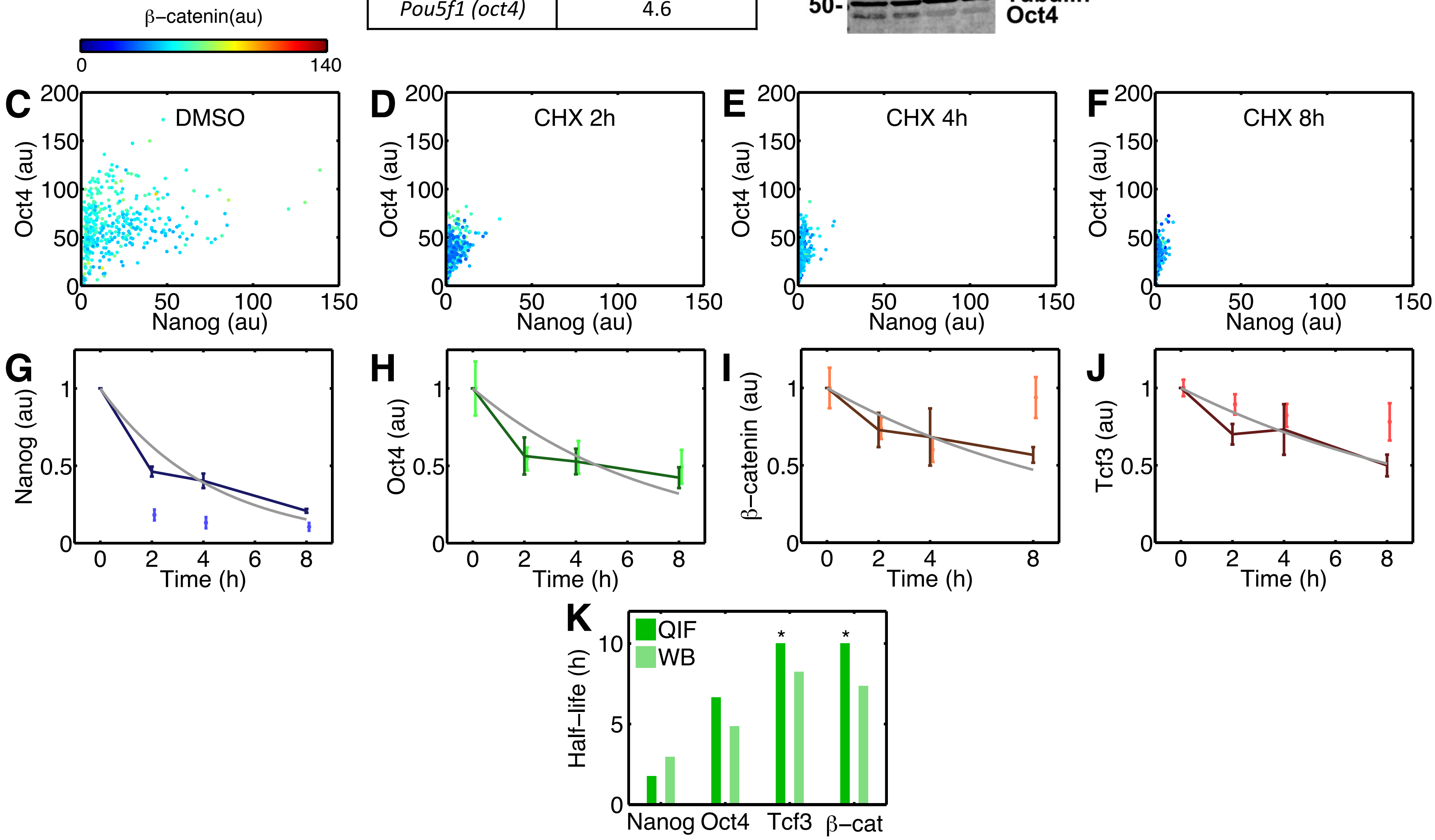

Fig. S4: A minimal competitive protein interaction network: The NOC model

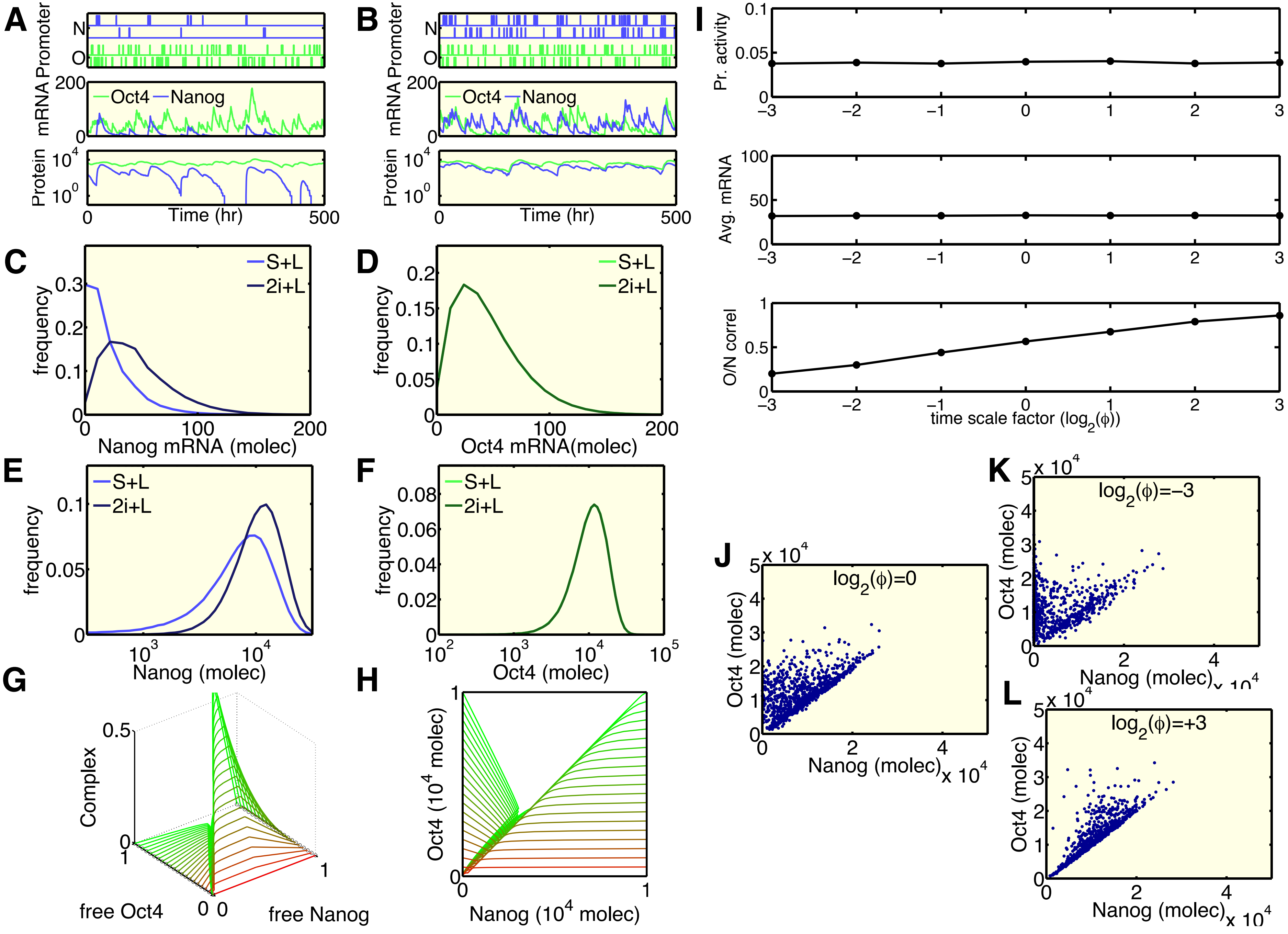

Fig. S5: Generalisation of the NOC model to multimeric interactions

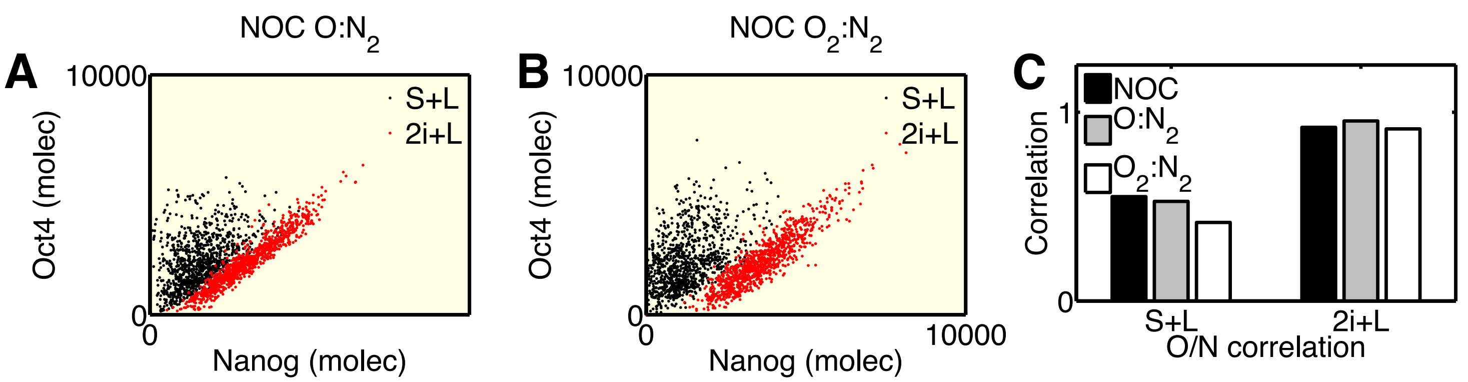

Fig. S6: Details on the TBON model

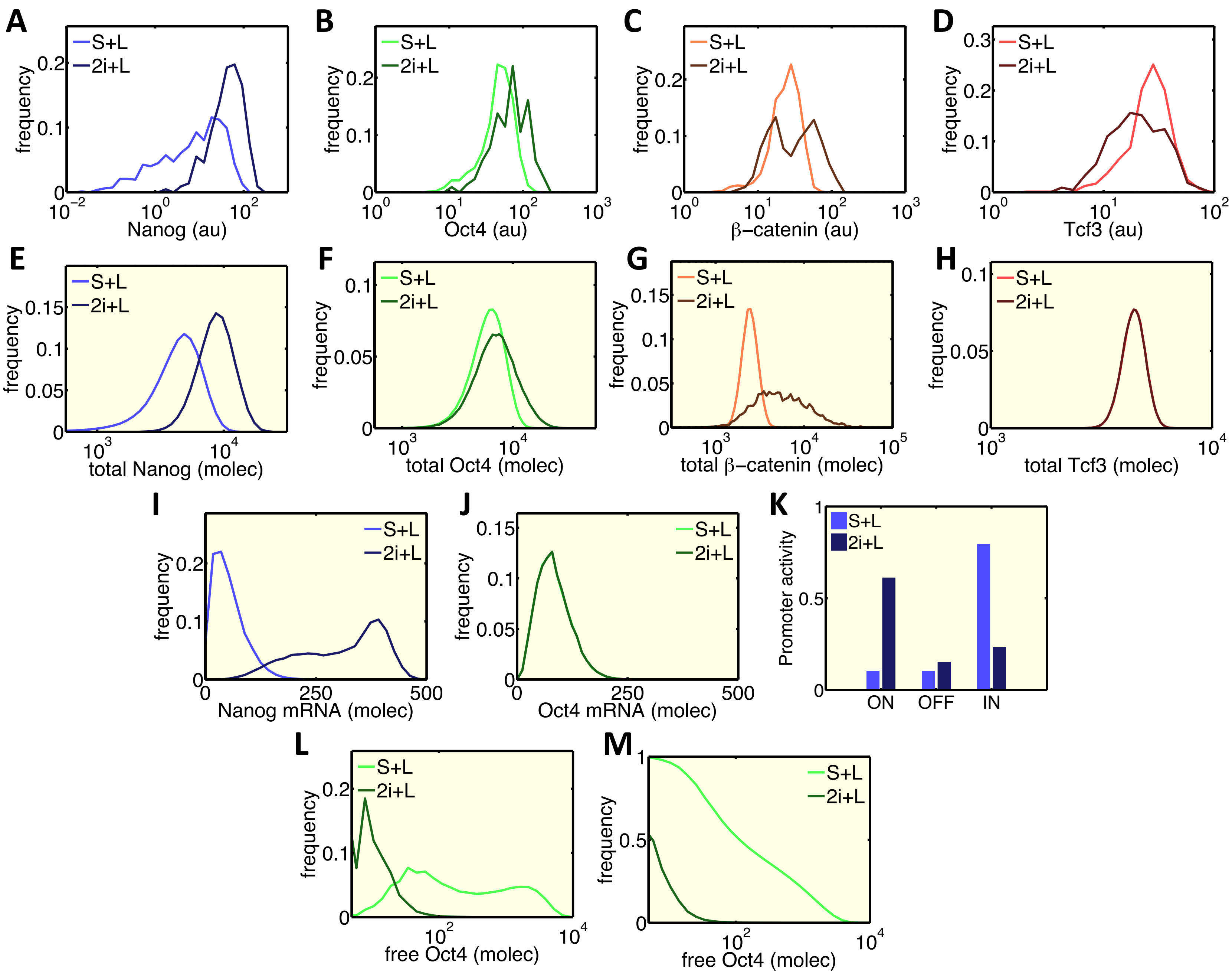

Fig. S7: Parameter sensitivity analysis of the TBON model

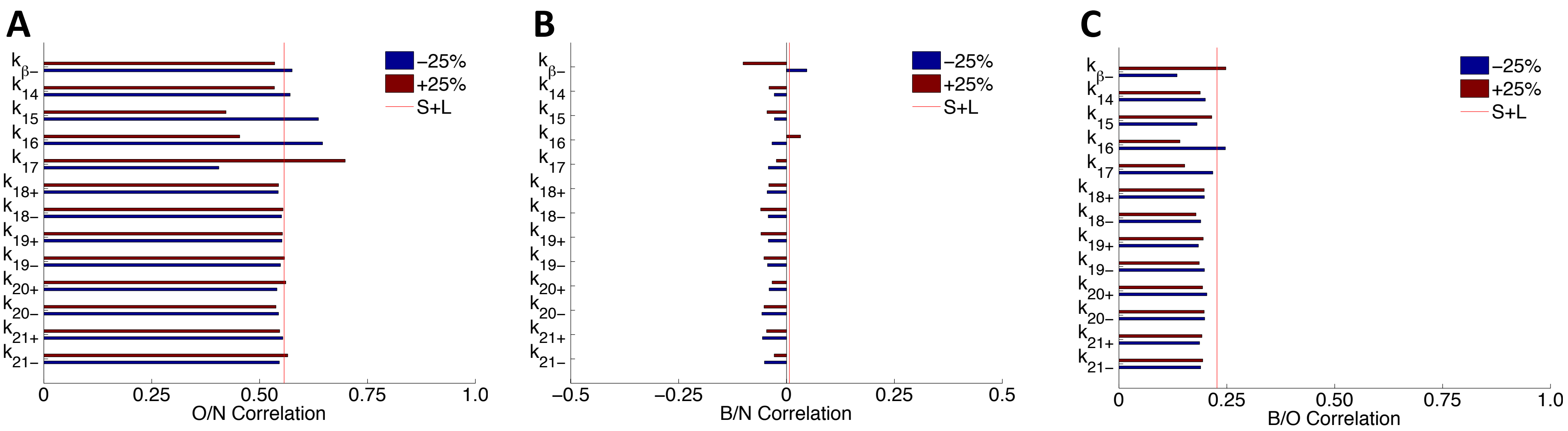

Fig. S8: Effects of Chiron and PD03 on the activity of the network

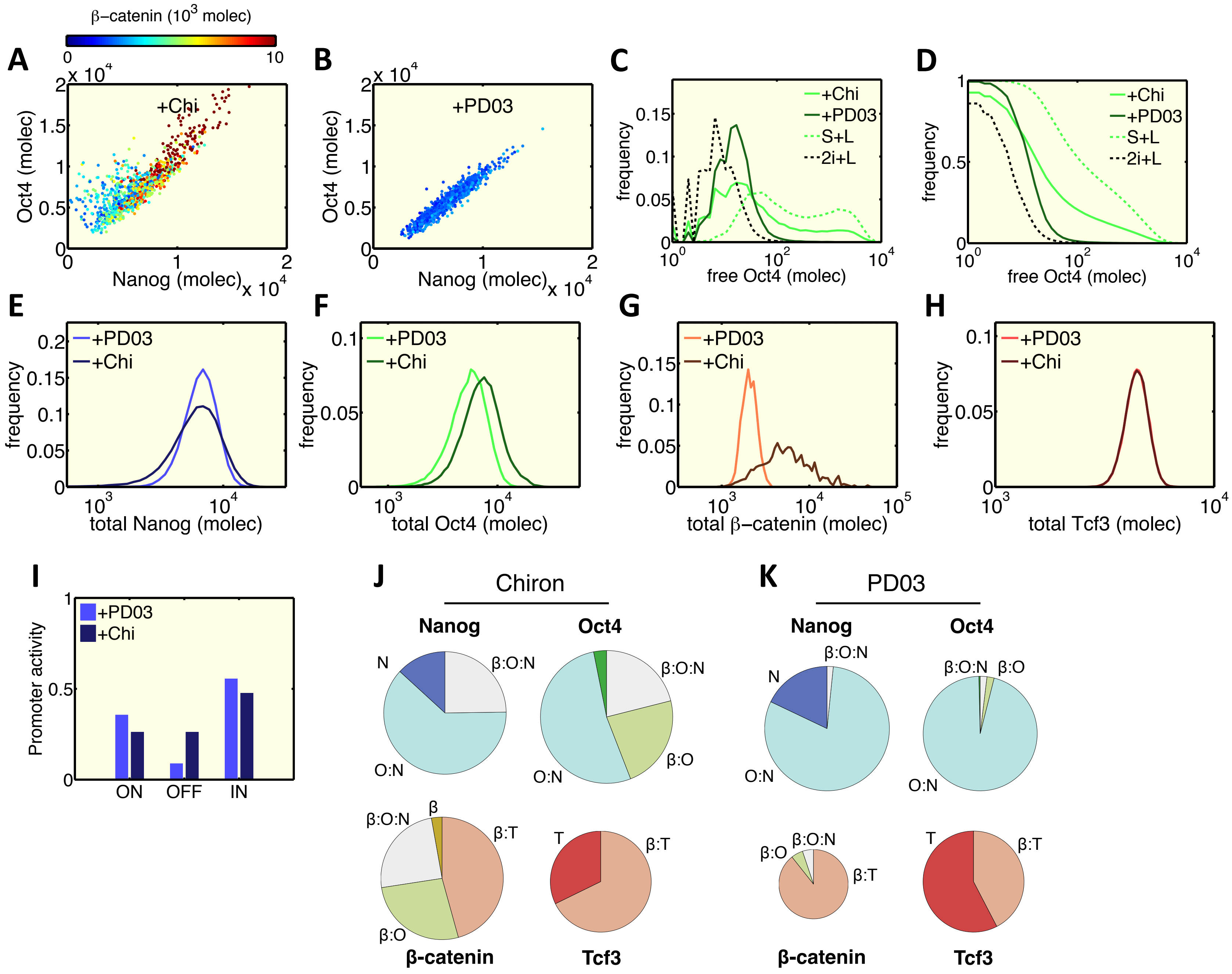

Fig. S9: TBON - Analysis of Oct4 and Nanog target genes

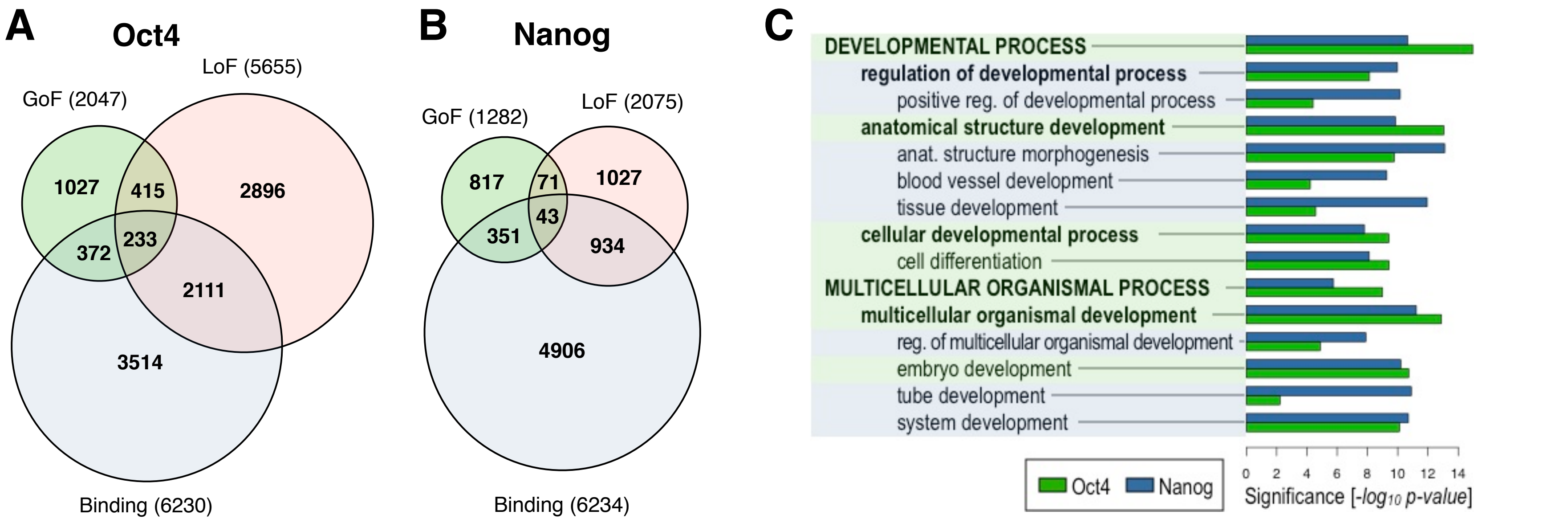

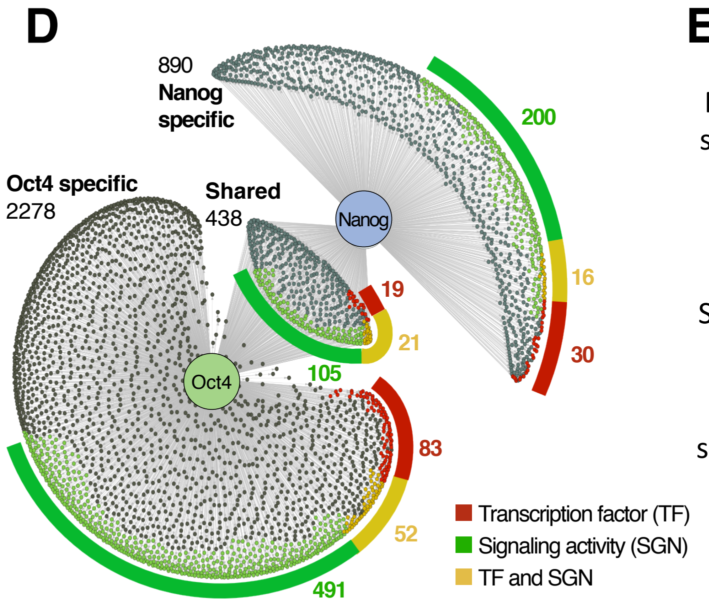

E

	Transcription factors				Signaling activity					
Nanog specifc	Cdx2	Gata3	Nfatc4	WT1	Adam17	Fgf1	Jag1	Mesp1	Rangap1	Wif1
	Ets1	Hes1	Pou2f3	Zfhx3	Adam23	Fgf15	Kit	Notch2	Rap2b	Wnt16
	Foxa2	Hoxb1	Stat1	Zic5	Bmp4	Fgf18	Kitl	Rab1b	Rassf1	Wnt3a
	Foxd3	Hoxb2	Stat6		Cdc42se1	Fgf3	Lats2	Rab27a	Rb1	213Wnt5b
	Foxk1	Hoxb6	Tbx20		Dock3	Fgf5	Lif	Rab34	Robo1	Wnt8a
	Foxn4	Myc	Tbx4		Dusp16	Fgfbp1	Map2k7	Rab8b	Sfrp1	
					Ephb4	Fzd7	Map3k3	Rab9b	Sfrp5	
					Fbxw11	Gja4	Map3k7	Ralgps2	Vegfa	
Shared	Evx1	Hoxa1	Runx1t	Stat4	Cul3	Fgf17	Gja1	Pdgfa	Vegfb	
	Gata6	Id3	Smad6	Zic2	Dab1	Fgf4	Gjc1	Sfrp2		
	Gli2	Pax6	Snai1		Dkk1	Fgfr2	Lrp5	Spry4		
	Hesx1	Rbpja	Snai3		Dusp6	Gata6	Nodal	Stk17b		
Oct4 specifc	Ctnnb1	Hoxc6	Nr5a2	Zic1	Adam11	Dock1	Map2k3	Nrp2	Rab20	Wnt10a
	En1	Hoxc9	Pax3	Zic3	Adam19	Fgfr1	Map2k5	Nrxn3	Rab2a	
	En2	Hoxd1	Pou4f2	Zscan10	Adam8	Fgfr4	Map3k10	Numbl	Rab40b	
	Etv1	Mef2d	Smad9		Akap2	Fgfrl1	Map3k11	Pdgfb	Rab7l1	
	Gata2	Meis1	Sox21		Akt1	Fzd10	Map3k7ip1	Pdgfra	Rab8a	
	Gli1	Meis2	Stat5a		Arf1	Fzd3	Map3k8	Pdgfrb	Rabif	
	Hey2	Myod1	Tbx21		Arf5	Fzd8	Mapk3	Pecam1	Racgap1	
	Hoxa13	Nfat5	Tbx3		Blnk	Jag2	Mapk4	Rab1	Ralb	
	Hoxa9	Nfkb2	Tead1		Bmp7	Jak1	Mapk8ip3	Rab10	Sfrs3	
	Hoxb9	Nkx2-4	Tead3		Cbl	Jak2	Mapk9	Rab12	Stk11	
	Hoxc4	Notch1	Zeb2		Ctnnb1	Jak3	Nkd1	Rab13	Stk38	
					Dll4	Map2k1	Notch1	Rab15	Vegfc	

Fig. S10: Stability of pluripotency in Nanog mutant cells

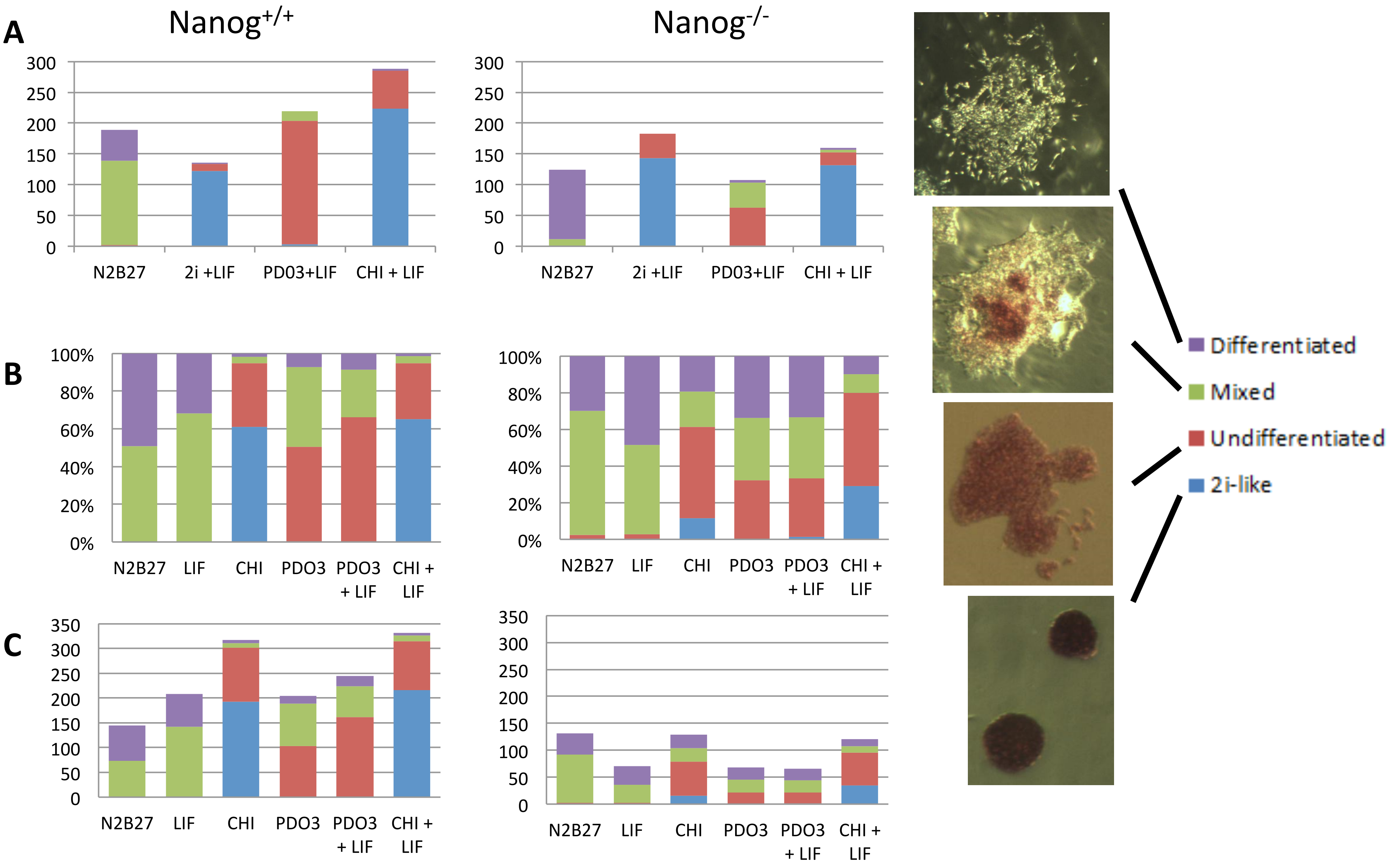

Fig. S11: Simulations of Nanog mutant cells in S+L and 2i+L

Nanog nulls in S+L

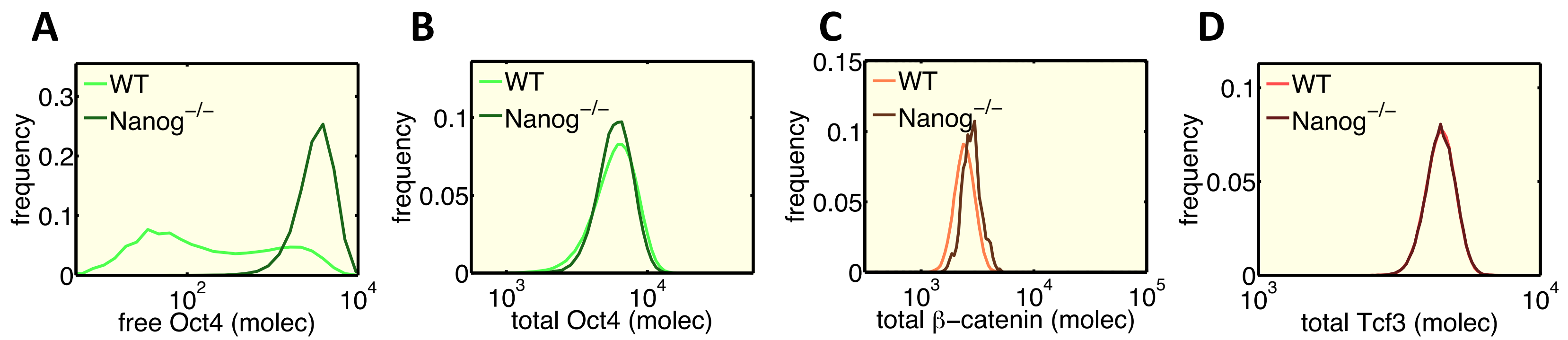

Nanog nulls in 2i+L

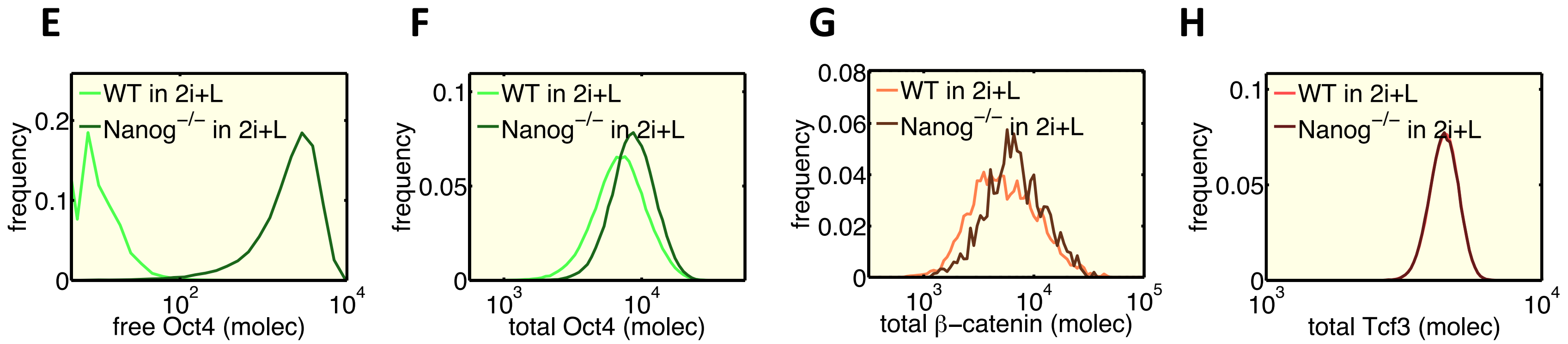

Fig. S12: Simulations of β-catenin mutant cells grown in 2i+L

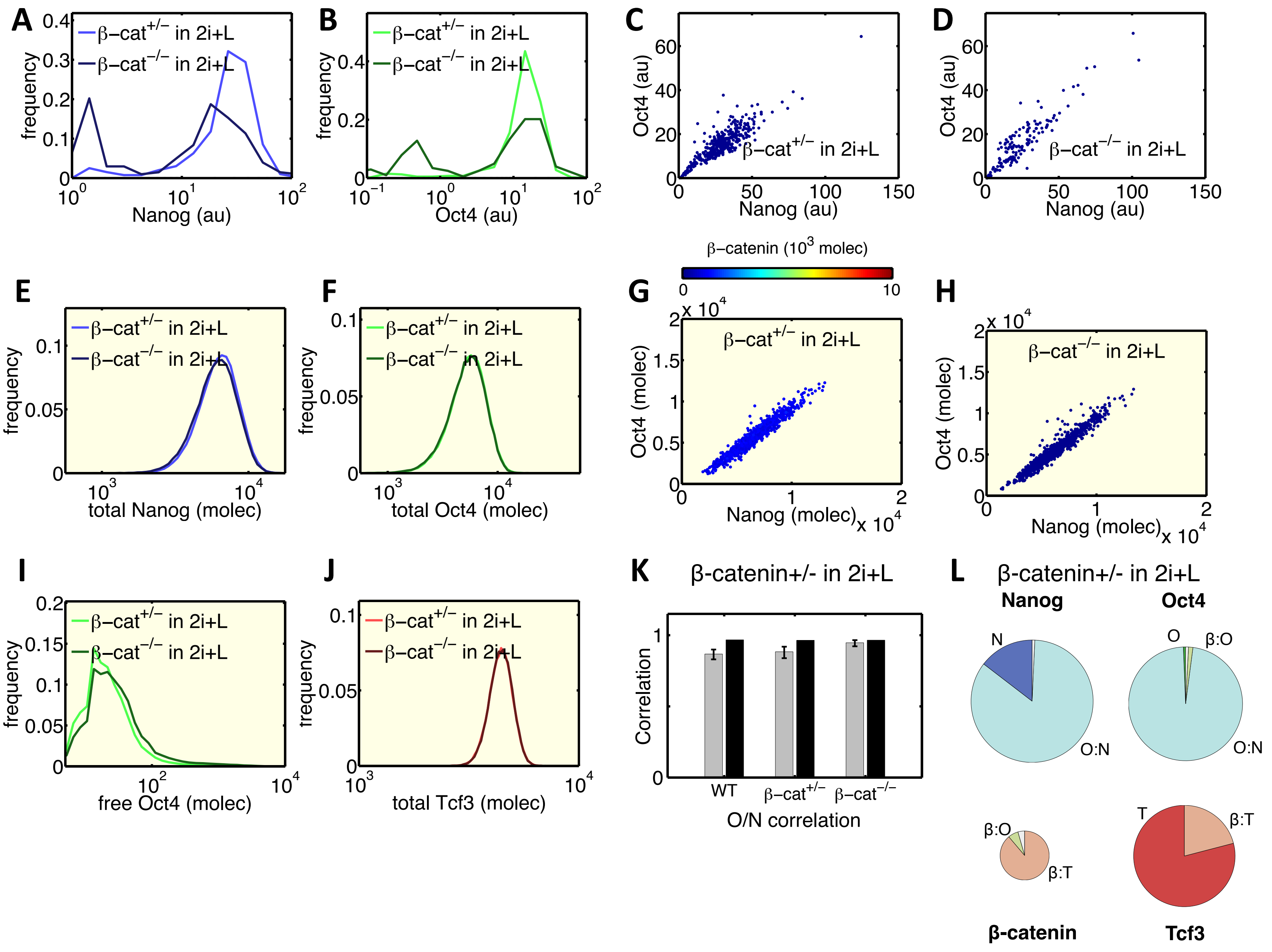

Fig. S13: Simulations of β-catenin mutant cells grown in S+L

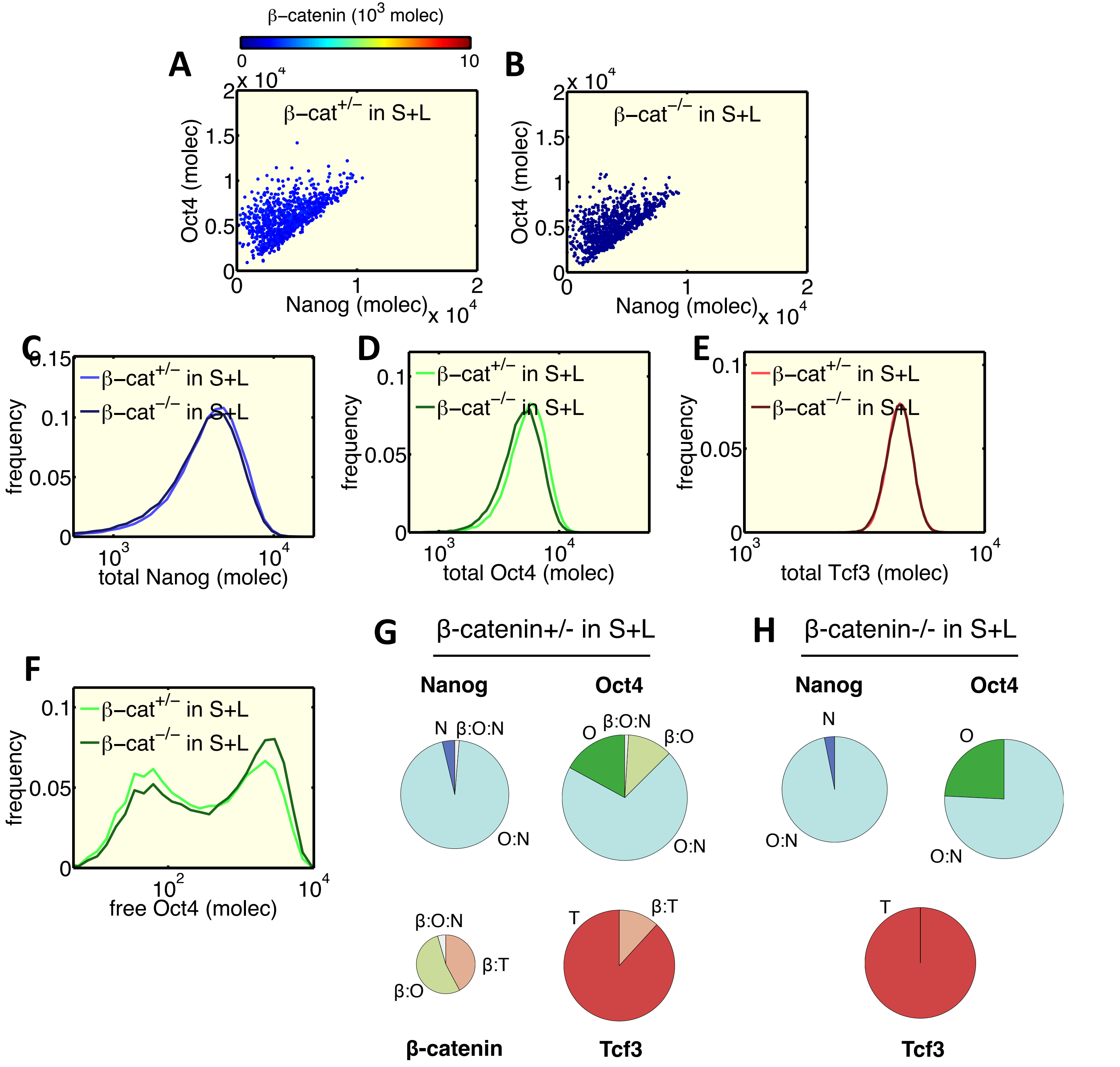

Fig. S14: Tcf3 mutant cells

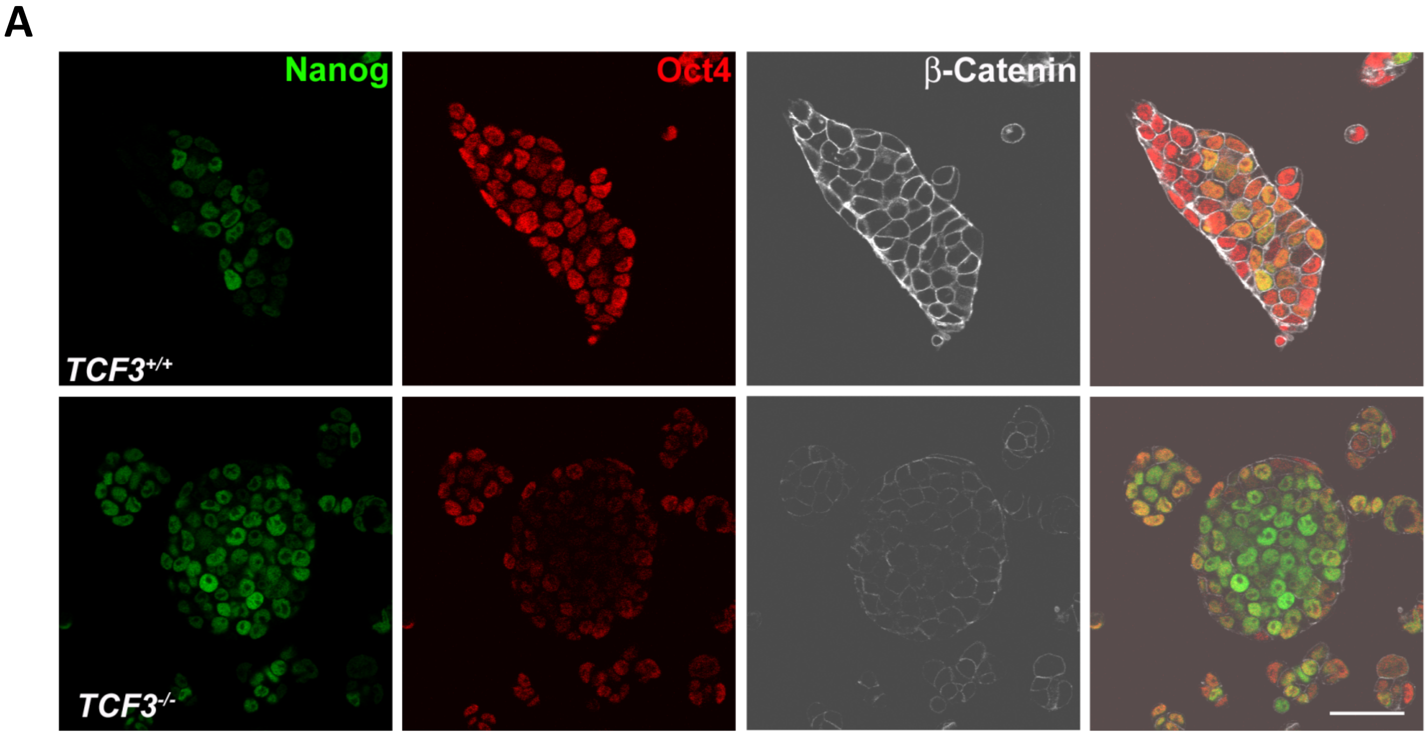

Fig. S14: Tcf3 mutant cells (cont)

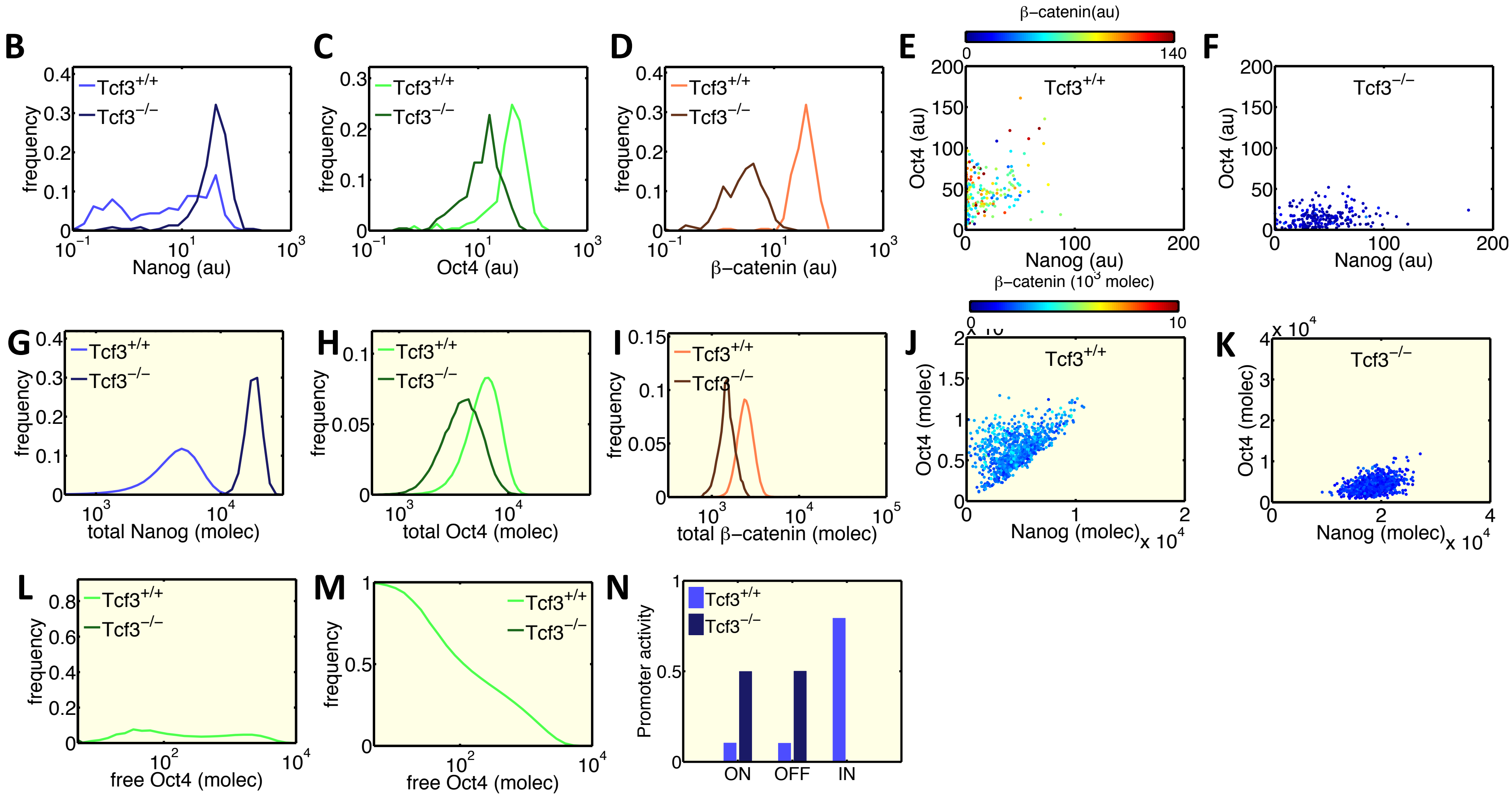

Fig. S15: Effect of Oct4 loss of function

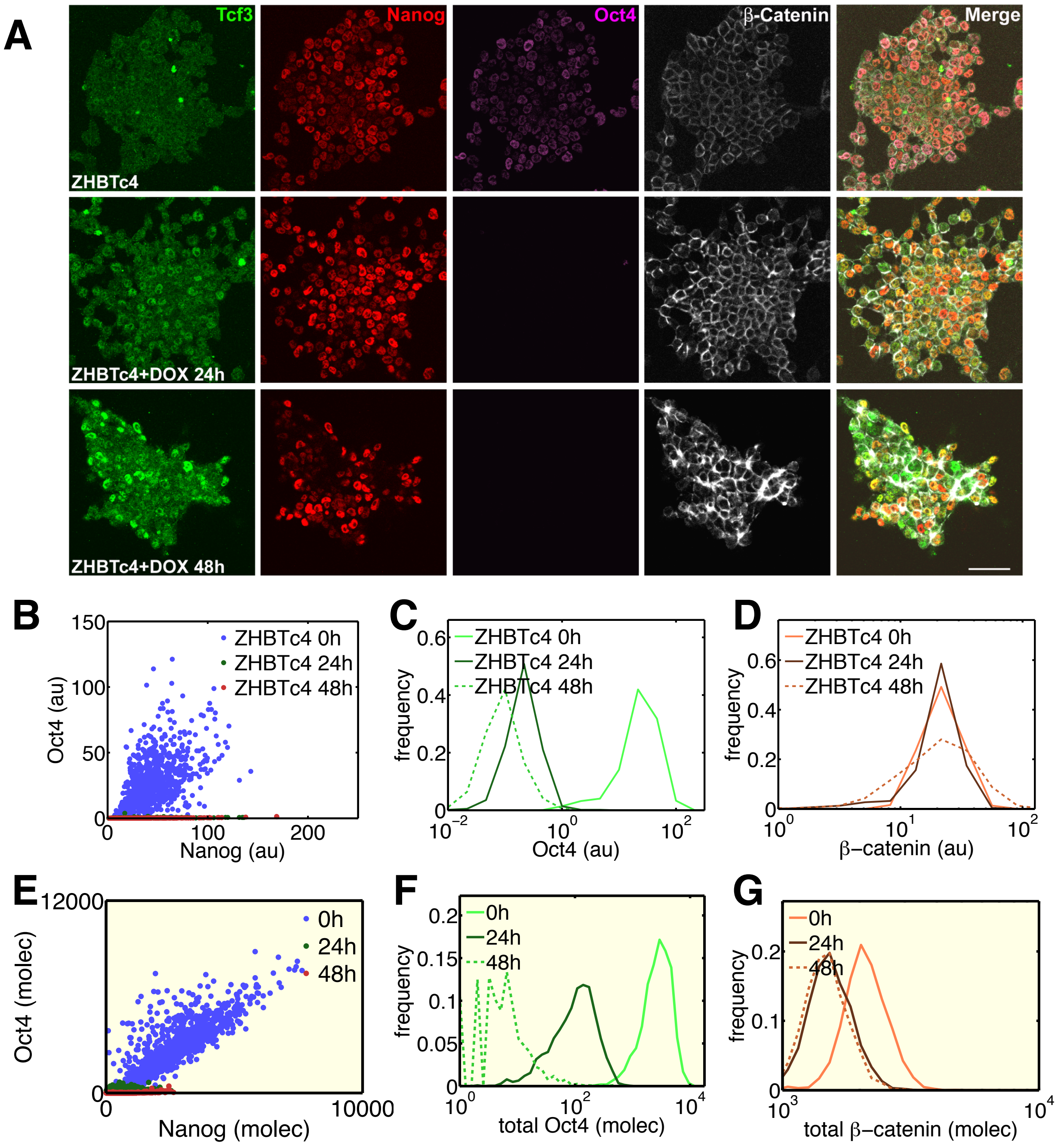

Fig S16: Effect of Oct4 overexpression

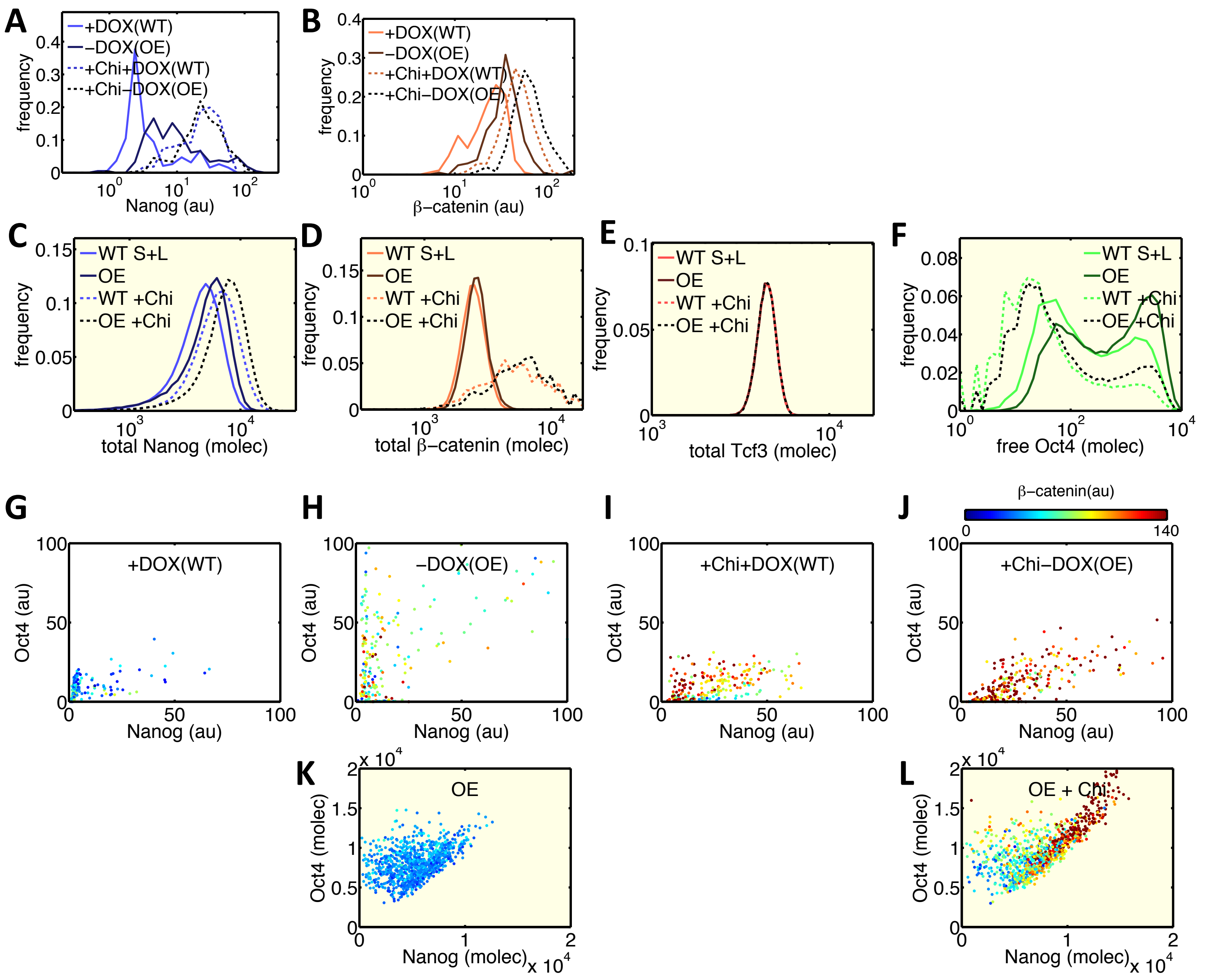

Fig. S17: Simulation of the decay of the TBON elements after cyclohexamide treatment

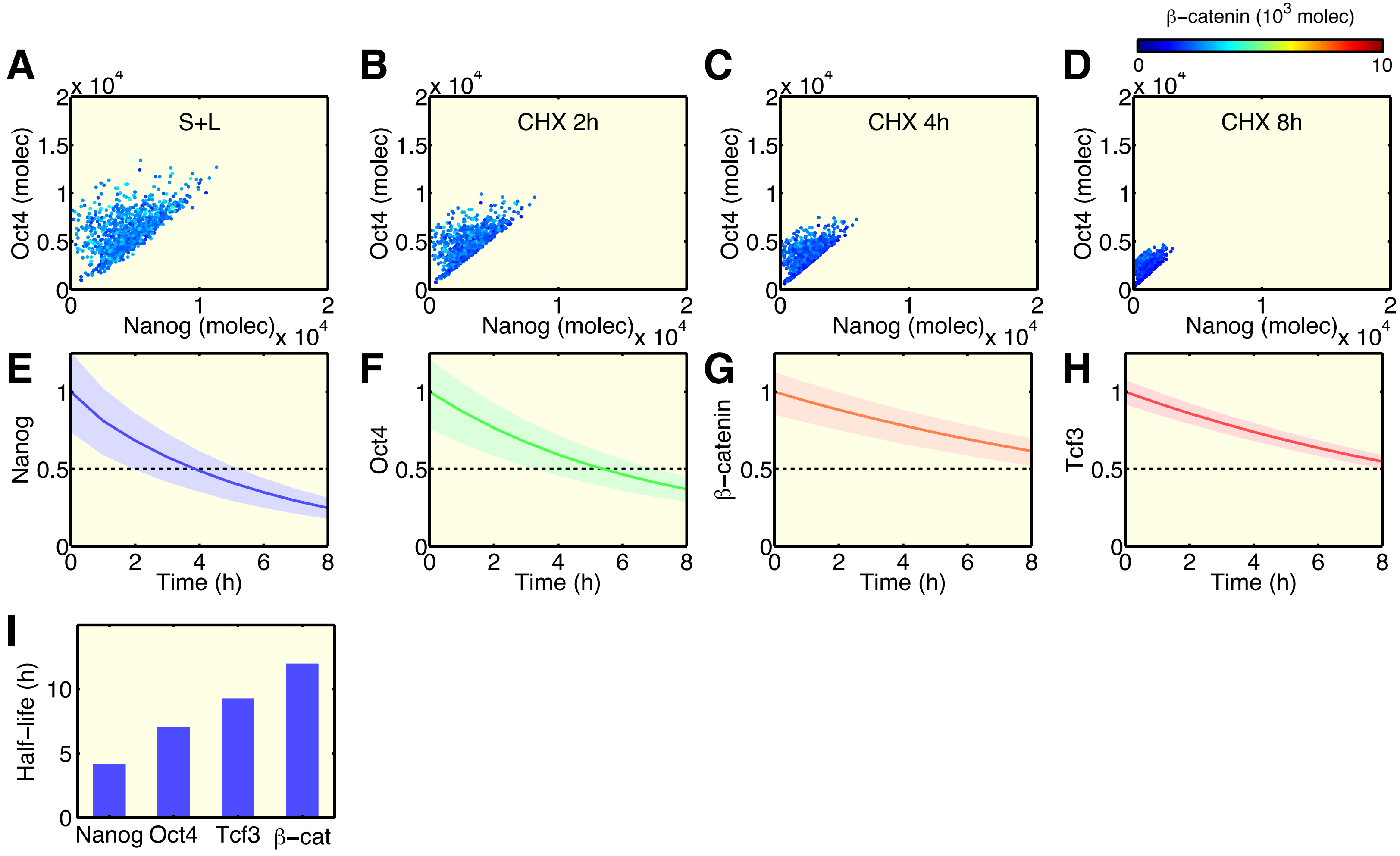

Fig. S18: TBON – Nanog self-repressive feedback loop

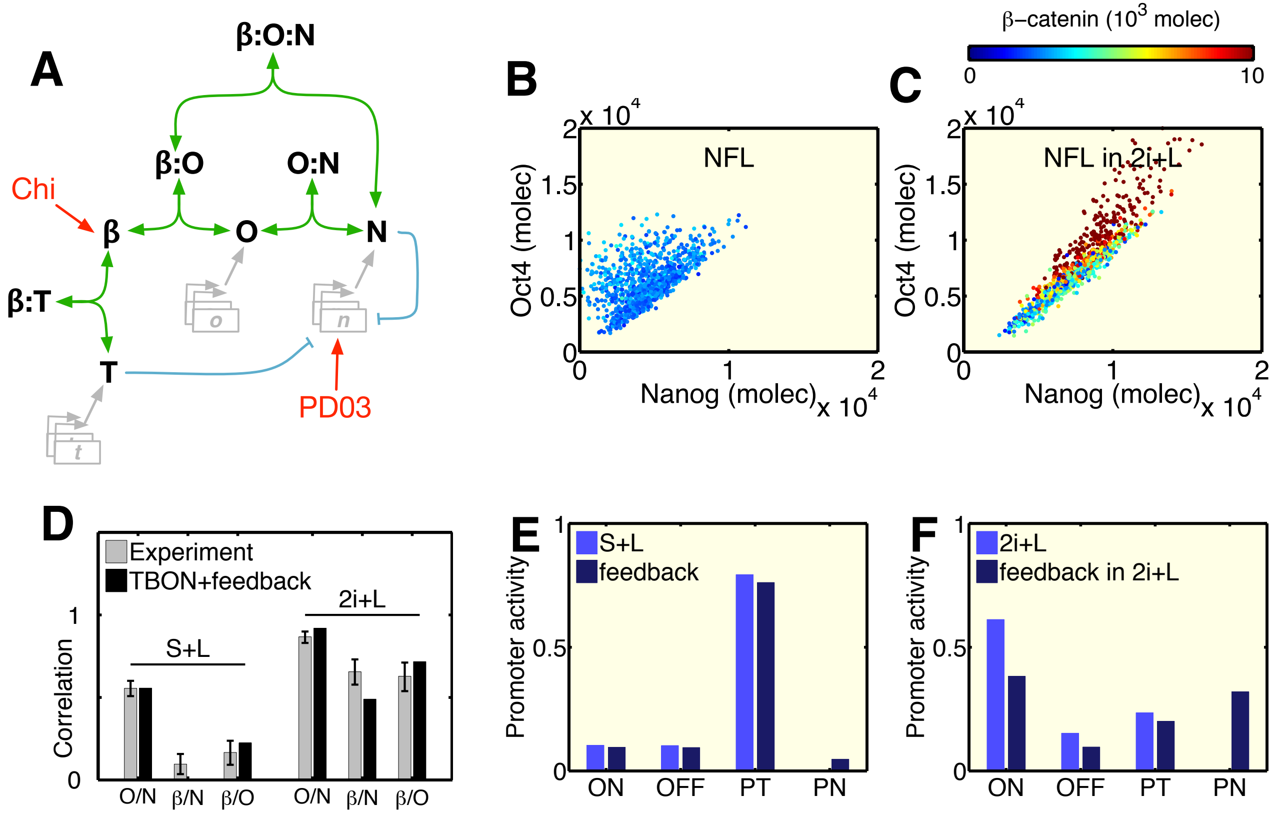

Supplement: Supplementary Information [file msb201349-s1.pdf]
